# Mass Spectrometry Proteomics of the Nanoparticle Corona Is Highly Dependent on Sample Preparation Protocol

**DOI:** 10.1002/pmic.70118

**Published:** 2026-03-27

**Authors:** Asia Saorin, Alberto Martinez‐Serra, Michael Henry, Paula Meleady, Marco P. Monopoli

**Affiliations:** ^1^ Chemistry Department Royal College of Surgeons in Ireland (RCSI) Dublin Ireland; ^2^ Life Science Institute Dublin City University Dublin Ireland; ^3^ School of Biotechnology Dublin City University Dublin Ireland

**Keywords:** biomolecular corona, mass spectrometry proteomics, nanoparticle corona, protein adsorption, sample preparation

## Abstract

It is widely established that the layers of proteins bound to the surface of nanoparticles (NPs), also known as the protein corona, critically influence their behavior in biological environments. Despite numerous proteomics studies performed in different NP‐corona systems, the impact of proteomic sample preparation methods on corona profiling remains poorly understood. In this study, we systematically compared five digestion protocols, namely S‐Trap^TM^, iST^TM^, ProteaseMAX^TM^, RapiGest^TM^, and a routine in‐gel digestion protocol commonly used in several laboratories. Protein corona samples were isolated from silica NPs incubated in 80% human plasma in phosphate‐buffered saline. Protocol performance was assessed in terms of digestion efficiency, protein and peptide yield, reproducibility, and identification bias. Proteomic characterization revealed marked differences across protocols, highlighting the influence of protocol on protein digestion, recovery, and representation. Our findings emphasize the need for method standardization and tailored protocol selection in corona studies to ensure accurate and reproducible proteomic profiling.

**Abbreviations**
ACN: acetonitrile
BCA: bicinchoninic acid (protein assay)
DCS: differential centrifugal sedimentation
DLS: dynamic light scattering
DTT: dithiothreitol
GRAVY: grand average of hydropathicity
HC: hard corona
HP: human plasma
IAA: iodoacetamide
IBD: in‐gel basic digestion
iST: isobaric‐stable‐tag (preomics) digestion cartridge protocol
LC‐MS: liquid chromatography–mass spectrometry
LFQ: label‐free quantification
microBCA: micro‐scale bicinchoninic acid
NP: nanoparticle
PBS: phosphate‐buffered saline
PCA: principal component analysis
PDI: polydispersity index
pI: isoelectric point
Pmax: ProteaseMAX (surfactant‐assisted digestion protocol)
QSAR: quantitative structure–activity relationship
RapiG: RapiGest (surfactant‐assisted digestion protocol)
RSD: relative standard deviation
SDS‐PAGE: sodium dodecyl sulphate polyacrylamide gel electrophoresis
SiNPs: silica nanoparticles
SPE / C18 SPE: solid‐phase extraction (C18 tip‐based)
S‐Trap: suspension‐trapping cartridge digestion protocol
TEM: transmission electron microscopy
TFA: trifluoroacetic acid
TIC: total ion chromatogram

## 1 Introduction

In recent years, the protein corona, formed when nanoparticles (NPs) are introduced into biological fluids, has gained attention as a critical determinant of NP behavior in biological systems [1]. This corona is typically described by two different layers [2]: first, a loosely bound, rapidly exchanging soft corona, and then a more stable hard corona (HC), formed by tightly bound proteins to the particle surface that majorly defines its biological identity [3]. Whether applied in drug delivery, diagnostics, or nanosafety evaluations, a clear and reproducible characterization of the NP‐protein corona is necessary to define the biological identity of NPs and to ensure consistent interpretation of their interactions with cells and tissues. For instance, high‐quality datasets are required to develop robust quantitative structure activity relationship (QSAR) models, which depend on validated, consistent data to produce accurate and reproducible predictions [4, 5, 6]. However, current literature presents contradictory findings regarding the protein corona, for example, in predicting NP–cell interactions. These discrepancies may arise from the specific NP and cell line combinations investigated, or from inconsistencies in the methodologies used to isolate and analyze the corona proteins. For instance, these inconsistencies have been highlighted in recent multicenter studies, which revealed that identical NP‐HC samples analyzed across different core facilities produced widely variable results due to differences in sample preparation, LC‐MS instrumentation, and database search parameters [7, 8, 9]. Another aspect deeply influenced by data obtained from NP‐HC characterization is the one involving NPs for biomarker discovery. Indeed, biological fluids, such as blood plasma, contain thousands of proteins with significantly different abundances. The plasma proteome is primarily composed of a few highly abundant proteins, such as serum albumin, Immunoglobulin G, and fibrinogen, among others. Furthermore, it also includes numerous low‐abundance proteins that may serve as valuable biomarkers, potentially indicating early stages of disease [10, 11]. However, the complexity in identifying and quantifying these low‐abundance proteins due to the high abundance of other proteins makes the detection of low‐abundance biomarkers challenging. Over the last few years, research has been trying to use the corona of NP systems to enrich low‐abundance biomolecules, acting as a potential biomarker‐enriching tool [12, 13, 14, 15].

**Significance of the study**

Proteomic characterization of the nanoparticle–protein corona underlies our understanding of nano–bio interactions, influencing applications in drug delivery, diagnostics, nanosafety, and biomarker discovery. However, despite rapid progress in the field, corona proteomics remains affected by major inter‐laboratory inconsistencies. While previous multicenter studies have focused on variability in LC–MS platforms and data processing, the impact of sample preparation protocols themselves — the very first step of the workflow — has not been systematically investigated.

This study provides the first comprehensive, side‐by‐side comparison of five of the most commonly used digestion protocols for corona proteomics, integrating complementary metrics including digestion efficiency, peptide yield, quantification reproducibility, protein abundance profiles, and protocol‐specific identification bias. The results show that the choice of protocol substantially shapes the determined corona composition, altering both qualitative and quantitative outputs. Consequently, studies relying on different sample preparation strategies may not be directly comparable, even when analyzing identical nanoparticle–corona samples.

By demonstrating that protocol selection is a major source of analytical variability, this work highlights a critical bottleneck for data harmonization in nanomedicine and nanosafety. These findings motivate the development of community‐accepted best practices and pave the way toward standardized and FAIR corona proteomics workflows.

Therefore, it is clear that reliable proteomics data is essential. Mass spectrometry (MS), particularly nano‐liquid chromatography (LC) coupled with tandem MS, is the cornerstone of protein corona analysis, offering a global, unbiased overview of the corona composition. MS‐based protein analysis follows two main strategies: bottom‐up, which analyses digested peptides [16, 17, 18], and top‐down, which examines intact proteins [19, 20, 21, 22]. In shotgun proteomics, a bottom‐up approach is applied to protein mixtures, and peptides are matched to theoretical spectra from a database to identify proteins. The bottom‐up approach is more widely used due to its relative simplicity and efficient peptide ionization, though it offers limited sequence coverage and cannot fully reconstruct entire proteins [23].

Several approaches are employed for the protein corona enzymatic digestion, namely in‐gel, in‐solution and on‐particles, each with distinct implications for data outcome [23]. In‐gel digestion is often used, but if only selected protein bands are excised, the analysis does not reflect the full corona composition [24]. Alternatively, in‐solution protocols involve the detachment of corona proteins from the NPs' surface using commercial precipitation reagents [25]. These methods, however, can suffer protein loss due to incomplete precipitation, poor solubilization, or handling errors during sample processing [26]. On‐particle digestion, in turn, performs tryptic digestion directly on the NM‐corona complex [27]. This approach minimizes sample handling, reducing the risk of protein loss, but introduces other challenges, such as the potential re‐adsorption of peptides onto the NPs' surface, and the limited access of trypsin to the proteins' cleavage sites within the tightly packed corona, potentially leading to incomplete digestion and lower sequence coverage [26].

Another point to consider in NP‐corona proteomics is the need to fully remove particles before injection into the nano‐LC to prevent column and system blockage. In the case of in‐gel digestion, the NPs remain trapped in wells of the gel, and therefore, no NP traces are likely to be found in the protein lysate. However, when other protocols are applied, the presence of NP must be carefully evaluated, and eventually, removal steps have to be introduced to avoid the introduction of NP into the LC‐MS instrument. Consequently, in‐gel digestion remains a reference method, particularly when gel electrophoresis is only partially run, allowing the sample to enter the separating gel without full migration. The entire protein‐containing region is excised [28], offering a practical compromise between sample cleanup, protein coverage, and workflow simplicity. Therefore, different aspects have to be considered and one of the main questions remaining in the field is whether MS‐based NP‐HC characterization is dependent on sample preparation protocols. One of the most critical steps in this workflow is protein digestion, where proteins within the NP corona are enzymatically cleaved into peptides for MS analysis. Variations in digestion protocols can significantly impact peptide recovery, enzymatic efficiency, and MS signal quality, ultimately affecting data reproducibility. Therefore, selecting an optimal digestion method is crucial for obtaining reliable proteomic data.

In this study, we compare the proteomics results obtained from five distinct NP‐corona sample preparation protocols. The primary aim of this study was to compare widely adopted, standardized sample preparation workflows that are compatible with routine LC‐MS proteomics and, importantly, those available from commercial platforms (e.g., cartridge‐based or surfactant‐assisted digestion systems). On‐particle digestion approaches were not considered since they are more dependent on nanoparticle physicochemical properties and are characterized by the previously discussed limits. Three out of the total five protocols consist of different variants of in‐gel digestion, namely the *in‐house* previously published protocol (basic in‐gel digestion, IBD) [28] and two other protocols (RapiGest^TM^, ProteaseMAX^TM^), where trypsin enhancer surfactants were added, and samples were treated as prescribed by manufacturers’ instructions, with a slight adjustment for the NP‐HC. The other two methods consist of commercial cartridge protocols (S‐Trap^TM^, iST).

Each digestion method offers some advantages and limitations. The S‐Trap^TM^ (Strap)and iST methods provide efficient protein trapping and digestion in confined environments, which are supposed to enhance peptide recovery and minimize sample loss, making them well‐suited for high‐throughput workflows. RapiGest^TM^ (RapiG) and ProteaseMAX^TM^ (Pmax) improve the solubilization of hydrophobic proteins and accelerate digestion, and their reliance on surfactants requires careful removal to prevent interference with MS analysis. Standard in‐gel digestion, while useful for targeted protein isolation and separation of isoforms, is time‐consuming and often results in lower peptide recovery compared to in‐solution approaches [26]. The choice of protocol depends on factors such as sample complexity, protein solubility, and the need for reproducibility in large‐scale studies. Therefore, analyzing the results derived from these methodological differences is essential for optimizing NP‐HC characterization and ensuring consistency in proteomic studies.

## 2 Materials and Methods

Silica particles (SiNPs), plain yellow green 100 nm in diameter, were purchased from Kisker (PSI‐GO.1).

The following reagents were purchased from Sigma Aldrich: Tris base (99%), acrylamide/bis‐acrylamide 40% solution, sodium dodecyl sulphate (SDS, 99%), ammonium persulphate, *N,N,N,N*‐tetramethylethylenediamine (TEMED, 99.5%), phosphate buffer saline (PBS) tablets and D‐(+)‐Sucrose (99.9%). One PBS tablet was dissolved in 200 mL of ultrapure water to obtain a 0.01 M phosphate buffer, 0.0027 M potassium chloride, and 0.137 M sodium chloride solution (pH 7.4 at 25°C). 3x blue loading buffer and 30X reducing agent (1.25 M DTT) were purchased from Cell Signaling Technology (USA). Imperial^TM^ Protein Stain and Pierce C18 Tips were purchased from Thermo Scientific Ireland.

The micro Bicinchoninic acid (BCA) assay kit was purchased from Thermo Fisher Scientific (TFS, Ireland). Human plasma (HP) from eight healthy donors provided by the Irish Blood Transfusion Service (IBTS) was mixed in equal proportions to obtain average pooled plasma. All the plasma sources were prepared from whole blood using the coagulant EDTA. The total protein concentrations were measured with BCA, following the manufacturer's instructions. Access and use of plasma samples were covered by the RCSI Ethics number 001246b.

S‐Trap^TM^ mini columns were purchased from PROTIFI, RapiGest^TM^ SF was purchased from Waters, and the iST kit was kindly provided by PREOMICS. ProteaseMAX^TM^ Surfactant was purchased from Promega.

**FIGURE 1:**
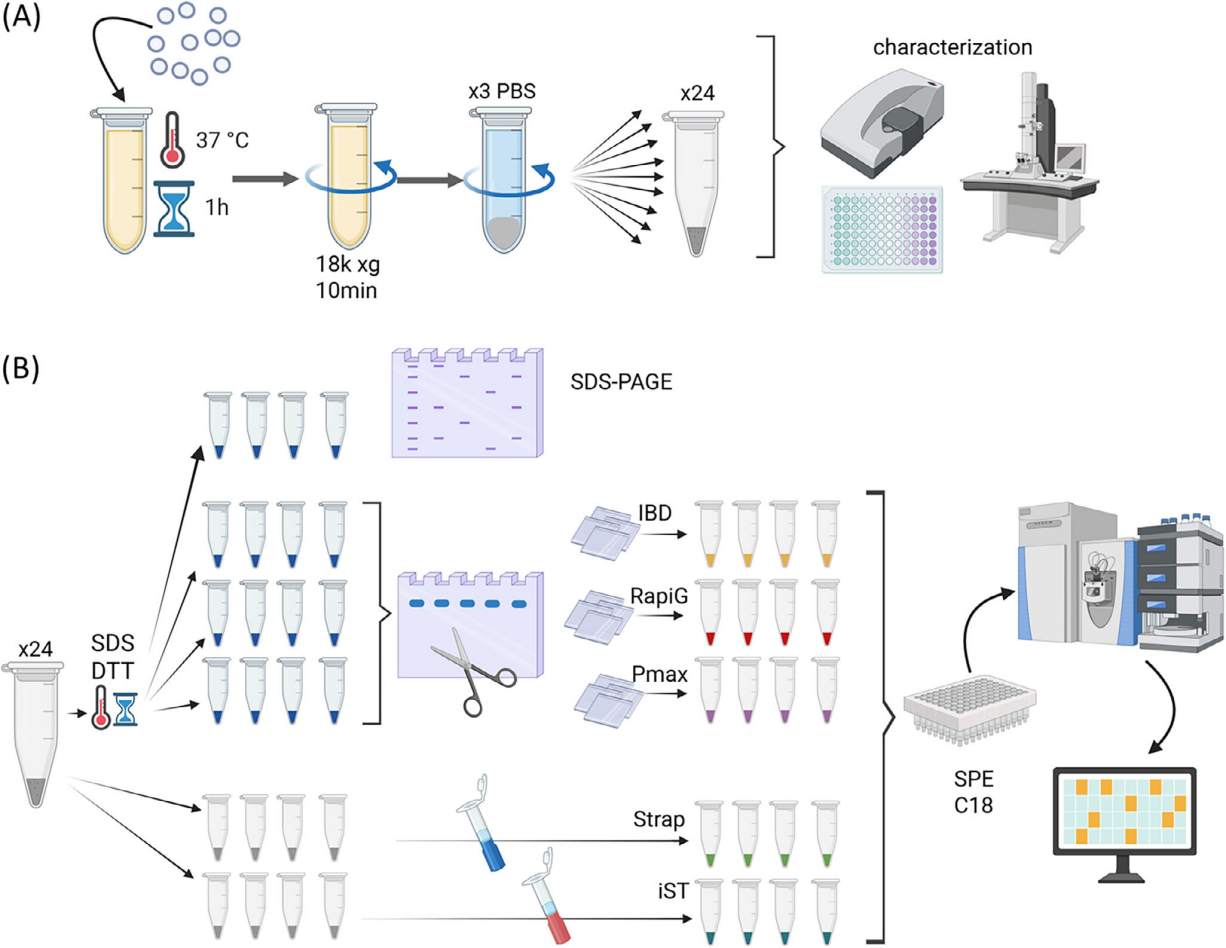
Workflow of sample preparation and analysis. (A) Isolation of HC‐SiNPs: SiNPs were incubated in an 80% human plasma solution, then subjected to sequential cycles of redispersion and centrifugation to obtain the final HC‐SiNPs sample. This sample was subsequently divided into 24 aliquots and characterized. (B) Proteomic analysis of HC‐SiNPs: Sets of four HC‐SiNP aliquots were subjected to SDS‐PAGE gel electrophoresis or processed using five different protocols, three based on in‐gel digestion and two utilizing cartridge‐based methods. All samples were then subjected to C18 solid phase extraction and analyzed using the same mass spectrometry set‐up.

Protein corona complexes of SiNPs (HC‐SiNPs) were obtained as previously reported [28, 29] by incubating 1 mg/mL of SiNPs in 2 mL of 80% human plasma in phosphate‐buffered saline (PBS). After incubation at 37°C for 1 h at 300 rpm in the thermoshaker, the samples were centrifuged at 18,000 xg for 10 min. The supernatant was then carefully removed without disturbing the pellet, and 0.4 mL of PBS was added. The pellet was resuspended by pipetting. The samples were subjected to an additional centrifugation‐redispersion cycle. The final sample was redispersed in 1.2 mL of PBS and divided into 24 aliquots, with PBS added to each to reach a final volume of 0.4 mL, followed by one more centrifugation step. After removing the supernatant, the HC‐SiNPs were stored at −20°C and thawed on the day of analysis. Four samples were used in each evaluated protocol and resuspended as reported in each section. Two batches of HC‐SiNPs were prepared simultaneously with the same aliquot of human plasma. Samples obtained from one batch were subdivided between the evaluated protocols and SDS‐PAGE analysis, while samples deriving from the other batch were used for colloidal stability evaluation and protein quantification (Figure 1) with micro BCA following a method described in Martinez‐Serra et al. [30].

### 2.1 Nanoparticle Characterization

Dynamic light scattering (DLS) measurements were performed using Zetasizer Nano ZS (Malvern). The sample cuvettes were equilibrated at 25°C for 90 s. Each measure's number of runs and duration were automatically determined and repeated three times. Differential centrifugal sedimentation (DCS) experiments were performed with a CPS Disc Centrifuge DC24000, using the standard sucrose gradient 8%–24% (Analytik Ltd.). A 544 nm PVC calibration standard was used for each sample measurement. Transmission electron microscopy (TEM) images were obtained with a JEOL JEM‐1400PLUS transmission electron microscope operating at a voltage of 120 kV. The statistical analysis was carried out using ImageJ, and the average size and standard deviation were measured on 103 NPs.

### 2.2 Micro BCA

The micro BCA assay was conducted to measure the amount of protein in HC‐SiNPs using the Micro BCA Protein Assay Kits, following the manufacturer's instructions for the microplate procedure. To quantify the amount of protein in the corona, two samples of HC‐SiNPs were resuspended in 450 µL of PBS. BSA standards with concentrations ranging from 200 µg/ml to 0.5 µg/ml were used for the calibration curve. 150 µL of Micro BCA working reagent was added to 150 µL of all samples in triplicate—standards, corona samples, and NP controls (pristine SiNPs in PBS)– and were incubated at 37°C for 2 h. Absorbance at 562 nm was measured using a Tecan Infinite 200 Pro microplate reader for both procedures. SiNPs' pristine absorbance values were subtracted from HC‐SiNPs.

### 2.3 SDS‐PAGE

SDS‐PAGE was performed as a quality control step to verify the consistency of the protein corona across all HC‐SiNP aliquots before the application of mass spectrometry sample preparation protocols. To perform the SDS‐PAGE, the pellets were re‐dispersed in 12 µL of PBS and mixed with 6 µL 3× loading buffer (62.5 mM Tris–HCl pH 6.8, 2% (w/v) SDS, 10% glycerol and 0.01% (w/v) bromophenol blue), heated at 95°C for 5 min and loaded (10 µL) in 10% polyacrylamide gel (prepared in the lab). Prime‐Step Prestained Broad Range Protein Ladder (6.5–270 kDa) (Biolegend) was used as a molecular weight marker. Gel electrophoresis was performed with a tris‐glycine buffer on a Mini‐PROTEAN electrophoresis system (Bio‐Rad) at a constant voltage of 120 V until the proteins neared the end of the gel. The gels were stained with Coomassie blue staining following the manufacturer's guide. Gels were scanned using an Amersham Imager 600 (GE Healthcare Life Sciences), and densitometry was obtained by GelAnalyzer 19.1 (www.gelanalyzer.com, created by Istvan Lazar Jr., PhD, and Istvan Lazar Sr., PhD, CSc).

### 2.4 Mass Spectrometry

#### 2.4.1 SDS‐PAGE Run for In‐Gel Digestions

HC samples were redispersed and heated as described in the SDS PAGE section. The total volume of samples was then loaded into the SDS‐PAGE gel and run at 120 V for about 10 min or until the proteins entered the separating gel. During this phase, the NP remains embedded within the wells or in the separating gel. The gel was carefully transferred to a clean surface, where the glass spacers were removed, and 1 cm bands were excised using a gel cutter. The bands were then cut (see protocol details) and placed into clean 1.5 mL microcentrifuge tubes.

#### 2.4.2 In‐Gel Basic Digestion (IBD)

The IBD protocol refers to a previous publication [28] and it represents the simplest in‐gel digestion method, without the addition of any surfactants, and is therefore considered the basic method. Excise gel bands were cut into four pieces and transferred to 1.5 mL centrifuge tubes previously washed with acetonitrile. Then, 100 µL of 200 mM ammonium bicarbonate was added to each sample and shaken for 10 min at 37°C. The buffer is then removed and substituted with 100 µL of 200 mM ammonium bicarbonate/acetonitrile (2:3) and shaken for 10 min at 37°C. The solution is then removed, and 100 µL of 50 mM ammonium bicarbonate are added and shaken for 10 min at 37°C. The solution is removed, and 100 µL of acetonitrile are added and shaken for 10 min at 37°C. Then, 100 µL of 10 mM dithiothreitol (DTT) are added, and samples are incubated at 56°C for 60 min. The solution is then removed, and 100 µL of 50 mM iodoacetamide (IAA) are added and shaken for 30 min in the dark at room temperature. Then, 300 µL of 100 mM ammonium bicarbonate are added to each sample and shaken for 15 min at 37°C. The solution is then removed and substituted with 300 µL of 20 mM ammonium bicarbonate/acetonitrile (1:1) and shaken for 15 min at 37°C. The solution is then removed, and 100 µL of acetonitrile is added and shaken for 10 min at 37°C. After solution removal, 100 µL of trypsin solution in 50 mM ammonium bicarbonate (trypsin: protein w/w in the range of 1:20 to 1:100) are added, and the samples are incubated overnight at 37°C. After incubation, the solution is recovered and transferred to a new tube. Then, 100 µL of 30% acetonitrile/0.2% trifluoroacetic acid (TFA) is added to each tube and shaken for 10 min at 37°C. After incubation, the solution is transferred along with the previous aliquot, and 100 µL of 30% acetonitrile/0.2% TFA is added to the gels and shaken for 10 min at 37°C. Finally, this last aliquot is collected and dried out in a vacuum concentrator.

#### 2.4.3 RapiGest^TM^ (RapiG)

The suggested procedure for in‐gel digestion was followed with slight adjustments. Gels were cut into cubes with an area smaller than 1 mm^3^, washed with 60 µL of water, and left for 15 min. The supernatant was then removed, and 40 µL of 50% acetonitrile/water was added, mixed (10‐second vortex), and left for 15 min. After removing the supernatant, 60 µL of 100% acetonitrile was added, mixed, and left for another 15 min. The supernatant was removed again, followed by the addition of 40 µL of 0.1 M ammonium bicarbonate, which was mixed and left for 5 min before adding 60 µL of 100% acetonitrile, mixing, and incubating for 15 min. The supernatant was then removed, and the gel pieces were completely dried using a vacuum concentrator.

Next, 50 µL of 10 mM DTT in 0.1 M ammonium bicarbonate was added and incubated at 56°C for 45 min. After removing the supernatant, 60 µL of 55 mM iodoacetamide in 0.1 M ammonium bicarbonatewas added and incubated in the dark for 30 min. The dehydration and rehydration steps were repeated, starting from the first addition of 50% acetonitrile to the drying step.

Then, 40 µL of 0.1% RapiGest SF solution in 50 mM was added and incubated at 37°C for 10 min, followed by complete drying of the gel pieces. Subsequently, 30 µL of trypsin (12.5 ng/µL in 50 mM ammonium bicarbonate) was added, and the samples were incubated on ice for 45 min. Excess solution was removed, and 60 µL of 50 mM ammonium bicarbonate was added, followed by incubation at 37°C overnight.

The digestion protocol was followed by the suggested sample preparation for LC and LC/MS. Specifically, 100 µL of 0.5% TFA was added to the samples that had dried after overnight incubation. The samples were then incubated at 37°C for 45 min, followed by centrifugation (13,000 xg, 10 min, 4°C). The supernatants were carefully transferred to new microcentrifuge tubes and dried prior to the application of the C18 tip protocol.

#### 2.4.4 ProteaseMAX^TM^ (Pmax)

ProteaseMAX solution was prepared following the manufacturer's instructions, in order to obtain a 1% solution in 50 mM ammonium bicarbonate. Samples were treated following the in‐gel digestion protocol provided. Briefly, gel bands were cut into cubes with an area smaller than 1 mm^3^, and the pieces were put in a microcentrifuge tube. The gel pieces were washed in 200 µL water and vortexed for 30 s, then the water was removed. Then, 200 µL of methanol:50 mM ammonium bicarbonate (1:1 v/v) was added, and the samples were intermittently vortexed for 1 min, and then the solvent was discarded. The procedure was repeated with the same solvent mixture, then intermittently vortexed for 5 min in 200 µL of acetonitrile:50 mM ammonium bicarbonate (1:1 v/v) and then mixed and incubated for 30 s in 100% acetonitrile. After solvent removal, samples were dried in a vacuum centrifuge. For alkylation and reduction, the dried gel pieces were rehydrated in 100 µL 25 mM DTT in 50 mM ammonium bicarbonate and incubated at 56°C for 20 min. The supernatant was then removed, and 100 µL of 55 mM of iodoacetamide in 50 mM ammonium bicarbonate was added, and samples were incubated in the dark at room temperature for 20 min. The supernatant is removed, and the gel pieces are washed twice with a brief vortex in 400 µL water. Then, the samples were dehydrated in 200 µL acetonitrile:50 mM ammonium bicarbonate (1:1 v/v) for 5 min under intermittent vortexing and then mixed and incubated for 30 s in 100% acetonitrile. The samples were then dried in a vacuum centrifuge. Digestion is performed by resuspending dried gel pieces into 20 µL of 12 ng/µL trypsin in 0.01% ProteaseMAX Surfactant:50 mM ammonium bicarbonate containing trypsin for 10 min. Add another 80 µL of the same buffer and mix the samples gently. The samples are incubated for 1 h at 50°C. Then, the samples were centrifuged at 12,000 × g for 10 s. The supernatant containing peptides is pipetted into a new tube, and TFA is added to a final concentration of 0.5% to inactivate trypsin. Samples were then maintained in ice for 10 min and then centrifuged at 16,000 xg for 10 min, supernatants were moved to a clean centrifuge tube and stored at −20°C.

#### 2.4.5 S‐Trap^TM^ (Strap)

46 µL of lysis buffer (5% SDS, 50 mM ammonium bicarbonate) were firstly added to the HC pellet and bath sonicated 5 min. Then 2 µL of 120 mM DTT in water were added and samples were incubated for 15 min at 55°C. After that, 2 µL of 500 mM IAA in water were added, and samples were incubated for 10 min at room temperature in the dark. Finally, 5 µL of phosphoric acid 12% in water were added and mixed under vortex for 30 s. Prior to the loading into the cartridges, 350 µL of binding/wash buffer (tris, 90% methanol, pH 7.55) were added and vortexed for 10 s. The buffer was obtained by diluting 1 M tris7.55 pH 1:10 in methanol, following the manufacturer's instructions. As a precipitate of tris was observed, the solution was then centrifuged and used. Then samples were loaded into the cartridges and centrifuged at 4,000 xg for 30 s. Cartridges were then washed three times with 400 µL of binding/wash buffer removed by centrifuge (4,000 xg for 30 s and last 1 min) and then transfer to a clean tube for the digestion. During the digestion step, the required amount of trypsin should be in the ratio 1/10 (trypsin/protein) and with a minimum required amount of 10 µg each cartridge. In the HC sample, total protein was equal to 7 µg, therefore the trypsin was added respecting the 1/10 ratio but without considering the minimum required amount. Trypsin was diluted in 50 mM ammonium bicarbonate and 125 µL of this solution was added to each cartridge and samples were incubated overnight at 37°C. Elution was achieved by the addition of 80 µL of 50 mM Tris in water, followed by the centrifugation of the cartridges (4,000 xg, 1 min). This step was repeated with 0.2% formic acid in water and as with 50% acetonitrile/water. These three fractions were pooled and the samples were dried. At the end of the elution phase, the presence of NPs was not determinable by visual eye inspection. Once the samples were dried in SpeedVac, the presence of pellets of NPs was observed.

#### 2.4.6 iST^TM^ (iST)

The kit provides a series of buffers for protein digestion and peptide extraction. LYSE denatures and reduces proteins, preparing them for digestion. The DIGEST buffer contains a Trypsin/LysC mix for enzymatic protein digestion, which is resuspended by RESUSPEND, while STOP halts enzymatic activity after digestion, and the WASH buffers remove hydrophobic and hydrophilic contaminants. ELUTE facilitates the extraction of peptides from the cartridge, and LC‐LOAD prepares the peptides for loading onto a reversed‐phase LC column for mass spectrometry.

The sample preparation was carried out following the manufacturer's instructions with minor modifications. 50 µL of LYSE was added to the corona samples in a reaction tube and placed in a thermoshaker heating block set to 95°C with shaking at 1,000 rpm for 10 min. After incubation, samples were quickly spun down to collect droplets (RT, 10 s, 300 xg). Then, the samples were cooled down and transferred to cartridges. Then 50 µL of the DIGEST reagent (prepared by adding 210 µL of RESUSPEND‐BCT and mixing 500 xg, 10 min) was added to the reaction tube containing the lysed protein sample and incubated in a pre‐heated heating block at 37°C with shaking at 500 rpm for 1 h. Following this step, 100 µL of STOP buffer was added to the reaction tube. Precipitation may occur at this step. The tube was shaken at RT at 500 rpm for 1 min and mixed by pipetting up and down. The cartridges were centrifuged (3,800 xg for 1 min). Then 200 µL of WASH 1 was added to the cartridges, and the centrifugation step was repeated. Then, this step was repeated with WASH 2. To elute the peptides, 100 µL of ELUTE buffer was added to the cartridge and centrifuged as before, collecting the flow‐through in the collection tube. Then the step was repeated, and the flow‐through was pooled in the same collection tube. The  CARTRIDGE was discarded and the COLLECTION TUBE wasplaced in a vacuum evaporator set to 45°C until the sample was completely dry. The protocol prescribed to use the LC‐LOAD buffer for reconstitution, but in order to compare with the other protocols, the dry samples were subjected to the same C18 tips as described in the previous protocols. This step assures the elimination of NP, as despite being visible as left over in the cartridges, we could not be sure to have achived full particle retention.

### 2.5 Mass Spectrometry Analysis

Dried samples obtained after the application of the different protocols were redispersed in 100 µL of 0.1% TFA, vortexed for 30 s, sonicated for 5 min, and shaken at room temperature for 30 min. Then, samples are purified by C18 tips (Thermo Scientific, 87784), following the manufacturer's instructions and using 0.1% acetic acid in 50% acetonitrile in water for elution from the tips. Samples are dried again in a vacuum concentrator and resuspended in 40 µL of 0.5% acetic acid in 50% acetonitrile. Taking into consideration the presence of NPs, S‐Trap samples were subjected to further centrifugation (18,000xg, 15 min), and the supernatant was moved to a clean tube. However, no pellets were observed, confirming the removal of NPs by C18 tips.

Analysis was performed using LC‐MS/MS on a Dionex UltiMate 3000 nanoRSLC coupled in‐line with an Orbitrap Fusion Tribrid mass spectrometer (Thermo Scientific). In particular, the LC‐MS run time was over 60 min, utilizing a resolution of 120K for full MS and 15K for higher‐energy collisional dissociation (HCD) MS/MS, with a mass accuracy of 10 ppm for peptides and 0.02 Da for MS/MS spectra.

Data are then analyzed with MaxQuant (v. 2.6.7.0). Parameters were set in order to keep the search and quantification strict, but minimizing the error. The maximum number of modifications per peptide was set to 3. Variable modifications were set as separate for the first search. Trypsin was selected as the digestion enzyme for all the tested protocols except iST, which was set as a separate group having both Trypsin and LysC, allowing for a maximum of two missed cleavages. Fast label‐free quantification (LFQ) was disabled, while a minimum LFQ ratio count of 2 was applied. The “Match between runs” feature was enabled to improve identification across samples. All other parameters were maintained as default. After data processing, contaminants and reverse hits were removed (serum albumin, which was identified as a contaminant, was retained).

### 2.6 Data Analysis

Data processing and analysis are performed using Python (version 3.12.2) and the Pandas library (version 2.2.2), implemented via the Anaconda distribution (Anaconda Inc., Austin, TX, USA) and the online platform MetaboAnalysist (a web‐based, comprehensive platform for metabolomics data analysis, developed by Xia Lab, Montreal, Canada). Graph Pad Prism 4.0 (Graph Pad Software Corporation, San Diego, CA, USA) was used for graphing. Prior to PCA analysis, LFQ data were filtered for constant or single values across proteins, namely, absent proteins (LFQ equal to 0 for all samples) were removed (261 proteins). Subsequently, proteins showing more than 80% of missing values were removed. The remaining missing values were input as 1/5 of the minimum values of each variable. Finally, samples were normalized on total LFQ values, log10 transformed, and auto‐scaled.

## 3 Results

### 3.1 NP Characterization

**FIGURE 2:**
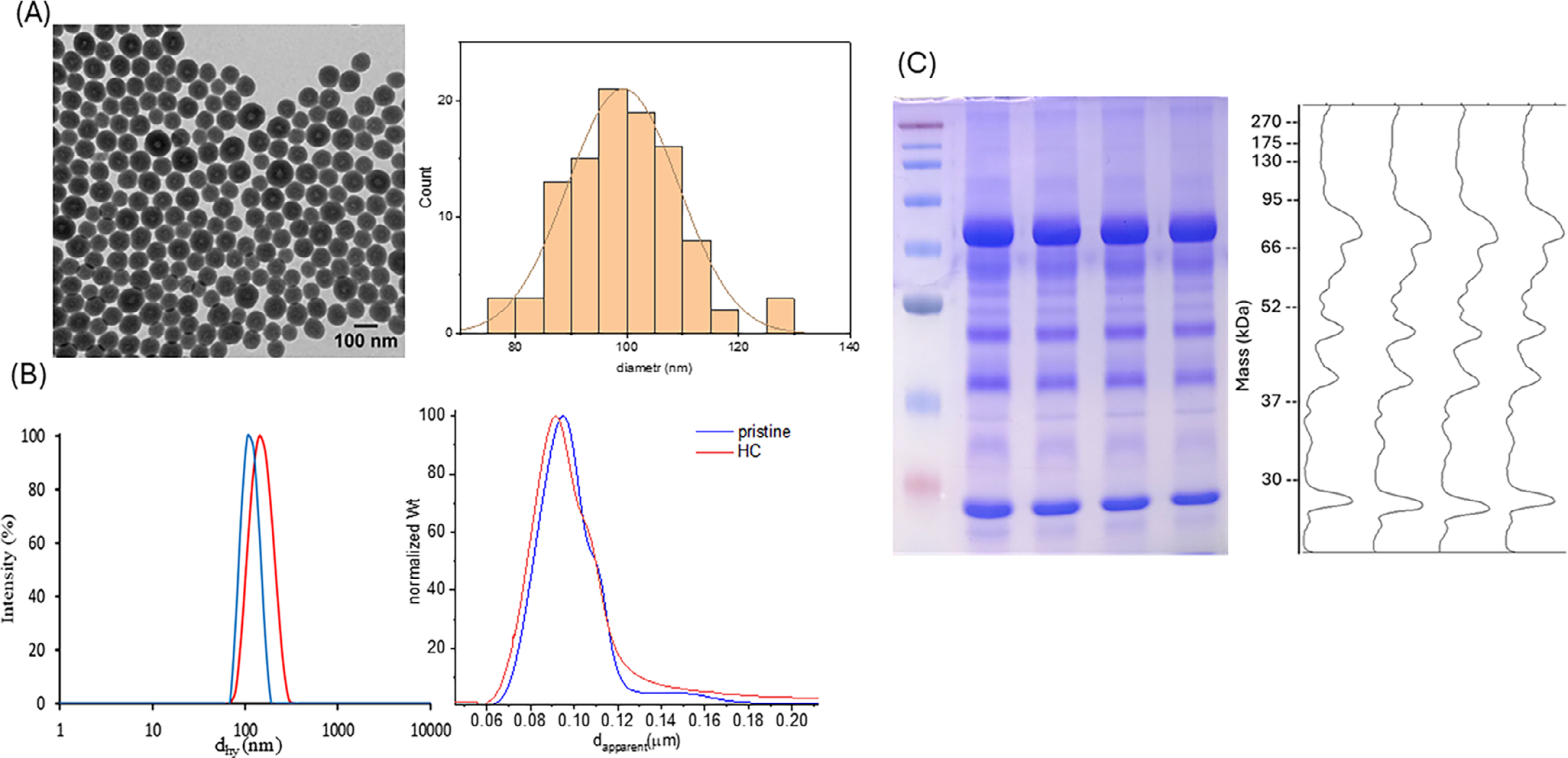
NP physicochemical and biomolecular characterization. (A) TEM image and size distribution of pristine NP. (B) DLS (left) and DCS (right) analysis of pristine NP and the NP‐HC. (C) SDS‐PAGE analysis of four replicates of NP‐HC, with gel image (left) and its densitometry (right).

Pristine NPs and NP‐HC complexes were characterized for size distribution, colloidal stability, and reproducibility (Figure 2). TEM analysis showed an average diameter of 99 ± 10 nm, while DLS measured a hydrodynamic diameter of 109 ± 1 nm for pristine NPs, increasing to 141.9 ± 0.8 nm for NP‐HCs. The polydispersity index (PDI) increased from 0.02 ± 0.01 to 0.05 ± 0.02, yet both values indicate monodisperse systems. DCS analysis supported the DLS results, with pristine and NP‐HCs showing sizes of 95.5 ± 0.4 nm and 93 ± 1 nm, respectively. The slight decrease for NP‐HCs reflects reduced overall particle density due to protein adsorption, without affecting dispersion, as indicated by consistent DCS profiles. SDS‐PAGE analysis of the NP‐HC samples consisting of the four replicates under study confirmed high reproducibility, showing consistent protein patterns. MicroBCA was then used for the quantification of protein corona, resulting in 29 ± 3 µg protein/mgNP.

### 3.2 Protocol Steps Comparison

The tested protocols differ significantly in handling complexity, time requirements, and suitability for NP‐containing samples (Supporting Information Protocol Table). The IBD, while time‐consuming (∼18–20 h), remains a reference due to its simplicity in terms of digestion, given that it just requires the use of only trypsin. In contrast, in‐solution methods like RapiG and Pmax streamline the process, with Pmax being the fastest (∼2–3 h). However, Pmax showed variability in sample volumes, likely due to the multiple vortexing steps that may not be highly reproducible between samples. In the case of the Strap protocol, several digestion options are available (see Supporting Information Protocol Table). We opted for overnight incubation at 37°C, as this temperature is the most commonly used across protocols. Consequently, the Strap digestion required approximately 18 h. A potentially critical aspect of this protocol was the inability to meet the minimum recommended trypsin‐to‐protein ratio for the cartridge, as the protein corona mass fell below the suggested working range. Notably, only the Strap protocol showed detectable NP presence following processing. The iST kit is among the quickest (∼3 h), with minimal hands‐on time and standardized buffers, though the trypsin concentration remains undisclosed and includes LysC. Trypsin gold was used in all protocols except for the iST kit, with enzyme‐to‐protein ratios ranging from 1:10 (RapiG, Strap) to 1:20 (Pmax) and 1:50 (standard in‐gel).

### 3.3 Protocol Quality: Digestion and Cleavage Efficacy

**FIGURE 3:**
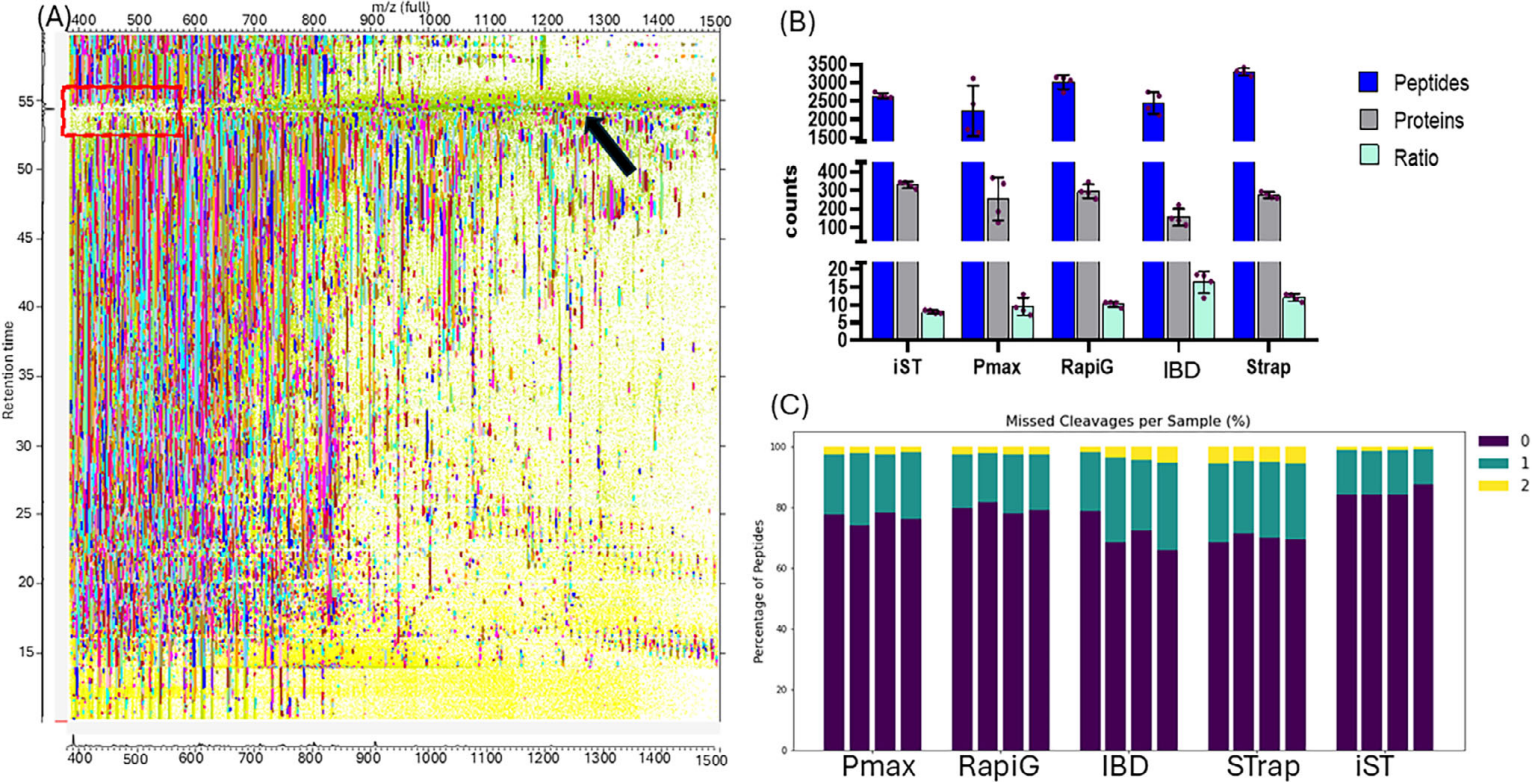
Proteins and peptides identification. (A) Heatmap of Pmax4, where color points represent the identified MS/MS peptides. The red box highlights the lack of identified MS/MS at low *m/z*, while the black arrow highlights the high signal obtained at high *m/z*. (B) Average number of peptides (filtered for MS/MS > 0) and proteins (filtered for LFQ > 0) identified in each protocol and their peptides/proteins ratio (error bars indicate standard deviation, SD). (C) Percentage of missed cleavages per replicate, grouped by protocols.

The inspection of the total ion chromatograms (TIC) can be a useful tool to evaluate the presence of poorly digested proteins [31]. From the TIC analysis (Figures S1–S5), intense peaks at the end of the chromatographic run are observed in all four IBD replicates, as well as in replicates 2 and 4 of the Pmax. The heatmap visualization reveals that, in correspondence with these peaks, there is an increased signal at high *m/z* values, accompanied by a lack of low *m/z* identified MS/MS, respectively, highlighted with the arrow and the box in Figure 3. This suggests that these signals may result from incomplete digestion, generating fragments too large to be detected as peptides. This is also consistent with the lower number of identified peptides and proteins in IBD, as well as the large variability among Pmax replicates. Indeed, Pmax2 and Pmax4 exhibited the lowest identification yields among the Pmax replicates (Table S1). To quantify the prevalence of high *m/z* species, the fraction of MS signal above *m/z* 800 in the 50–60 min retention time window was evaluated (Figure S6). Pmax, RapiG, and IBD exhibited similarly high fractions, indicating a substantial presence of large, poorly digested fragments. Strap and iST, in contrast, showed significantly lower fractions (0.42 and 0.28, respectively), consistent with more complete digestion.

The peptide‐to‐protein ratio was evaluated since a higher peptide‐to‐protein ratio reflects higher protein sequence coverage and can lead to greater confidence in protein identifications [32]. Strap and IBD showed the highest peptide‐to‐protein ratio, followed by RapiG and Pmax, while iST exhibited the lowest ratio (Table S2, Figure 3).

Missed cleavage values were lowest and most consistent across replicates for the iST kit, likely due to the combined use of trypsin and LysC. RapiG and Pmax followed, though Pmax showed slightly higher missed cleavage percentages, particularly in the lower‐performing replicates Pmax2 and Pmax4. Higher missed cleavage rates were observed in Strap and IBD protocols, Strap likely due to a lower trypsin‐to‐protein ratio, and IBD probably because the minimum required trypsin amount was not met.

### 3.4 Quality of Protein Quantification

**FIGURE 4:**
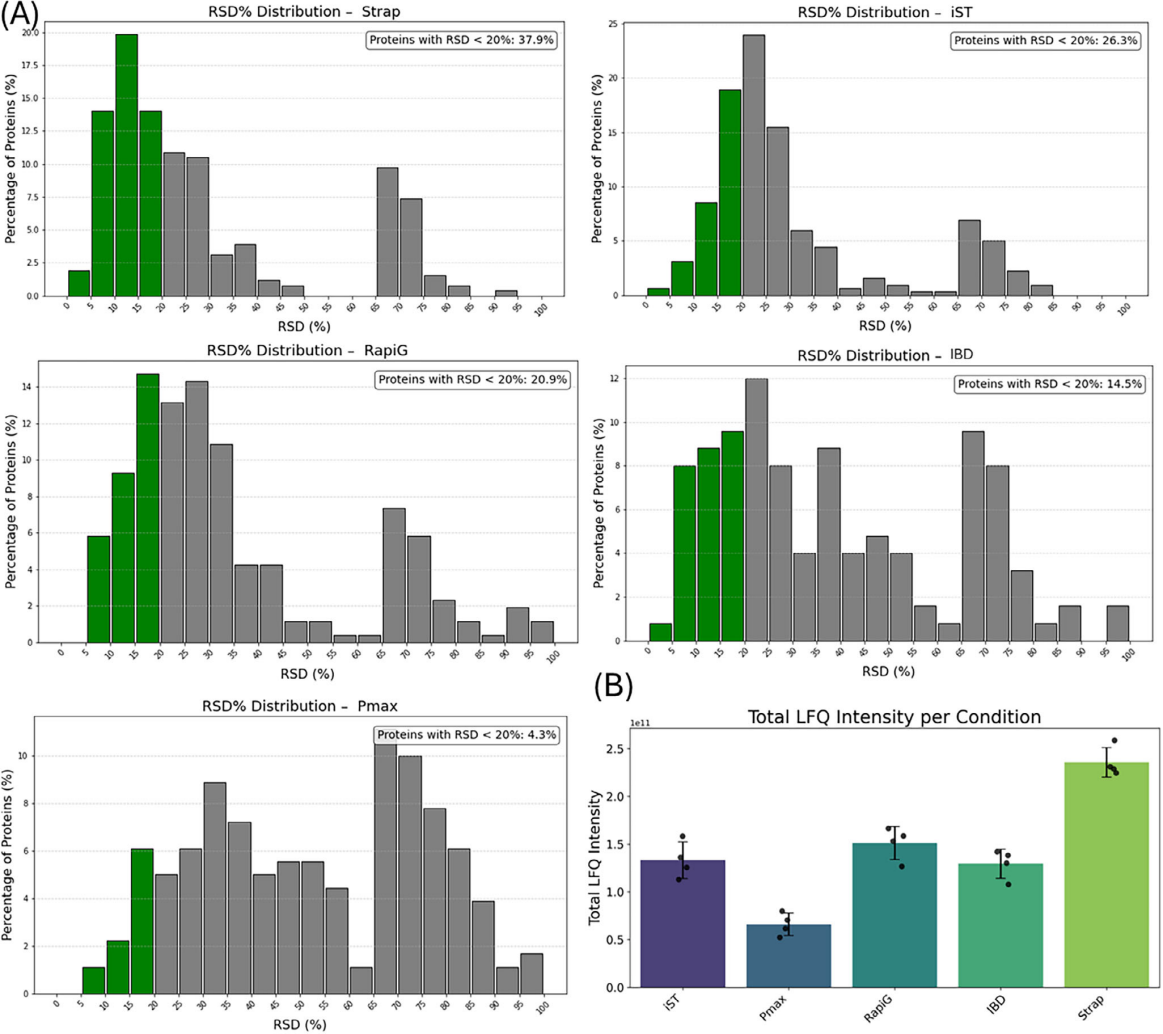
Reproducibility of peptide quantifications and total LFQ values. (A) Distribution of relative standard deviations (RSDs) calculated for each individual peptide. (B) Total LFQ values.

Across all protocols, approximately 45%–52% of identified proteins had LFQ values equal to zero, except for the IBD protocol, which showed a higher proportion of missing values at 67%. The distribution of relative standard deviations (RSDs), used as a measure of quantification reproducibility, revealed the presence of two main populations in all protocols: One group of proteins with RSDs below 50%, and another with substantially higher variability (Figure 4). The relative abundance of low‐variability (high‐quality) quantifications decreased in the following order: Strap, iST, RapiG, IBD, and Pmax. Notably, Pmax had a significantly lower proportion of proteins with RSDs below 20% (less than 5%), indicating reduced quantification consistency. The protocols followed the same ranking in terms of total LFQ, indeed overall SD values are similar among protocols, therefore the lower RSD can be attributed to the higher LFQ values (Figure 4).

### 3.5 Protein Identification and Quantification: Overlaps and Differences

**FIGURE 5:**
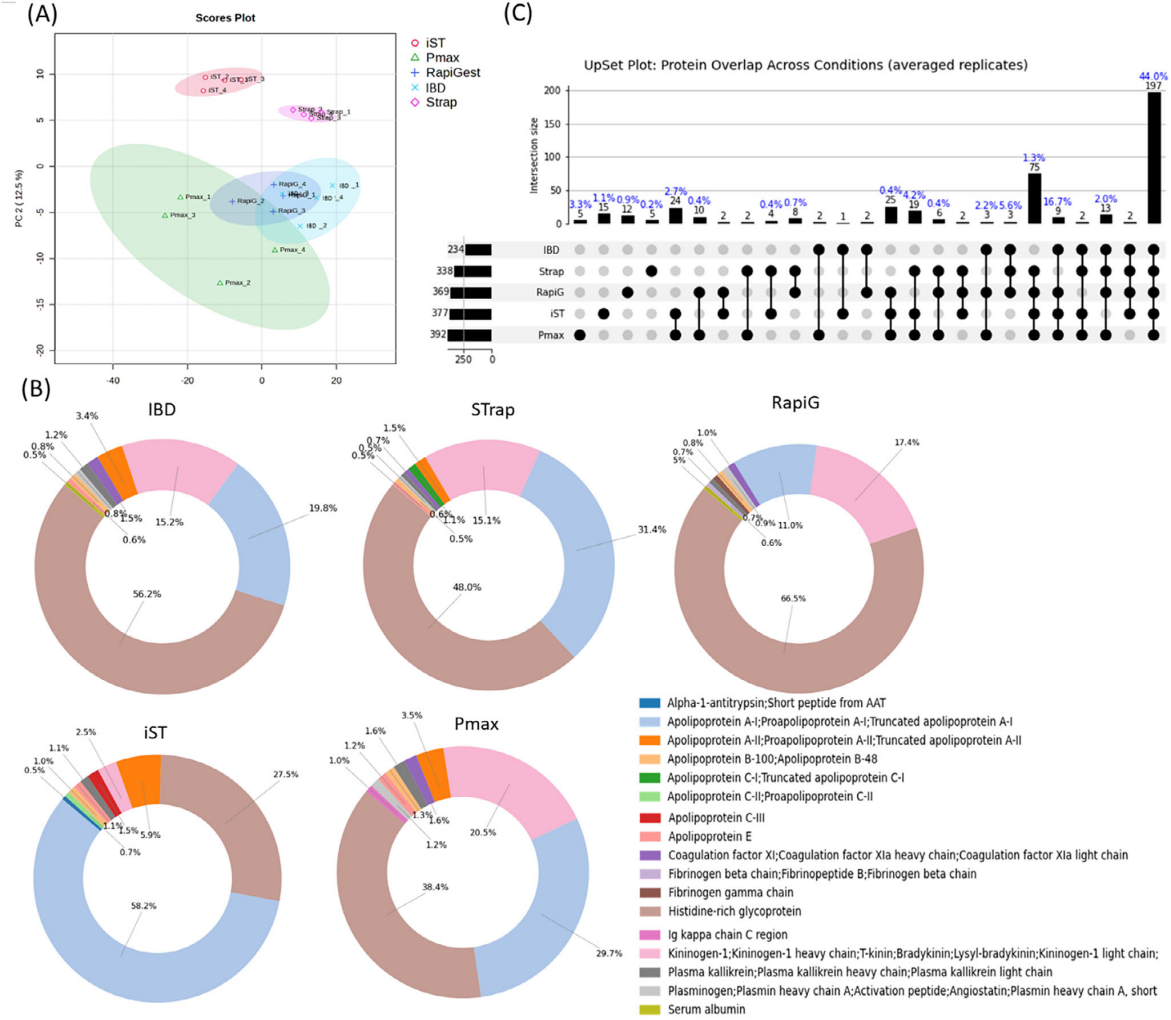
Summary of the proteomics analysis. (A) Principal component analysis (PCA) scores plot. (B) An UpSet plot shows how many proteins are shared or unique across different groups. The bars on the left show the total number of items in each individual group. The connected dots and bars at the top indicate the size of the intersections. LFQ average values were used. (C) Top 10 proteins identified in each protocol.

Principal component analysis (PCA) confirmed the lower precision of Pmax, as can be seen from the wider 95% confidence area and the separation of replicates 2 and 4 (Figure 5A). Moreover, it highlighted a higher similarity between in‐gel digestion protocols, given the separation from Strap and iST. From the biplot representation, we observe that the vertical separation is due to Apolipoprotein C‐IV (Figure S7). Histidine‐rich glycoprotein is the most abundant protein in all the protocols except iST, where it is the second most abundant, following Apolipoprotein A‐I (Figure 5B, Table S3), which reaches about 50% abundance on total LFQ intensity.

Histidine‐rich glycoprotein, Apolipoprotein A‐I, and Kininogen‐1 are the top three most abundant proteins in all the protocols except iST, being all above 10%, followed by Apolipoprotein A‐II, whose values significantly drop in the range 0.3%–3%. Differently, iST shows a higher content of Apolipoprotein A‐II followed by Kininogen‐1.

The relative abundance of Apolipoprotein A‐I was also estimated from SDS‐PAGE densitometry, where the intense band observed below 30 kDa in the SDS‐PAGE gel (Figure 1C) is attributable primarily to Apolipoprotein A‐I [33, 34]. Therefore, the area of this band was normalized to the total protein area in the densitometry profile, resulting in a value of 14.9 ± 0.9%. Although it is important to note that different proteins bind Coomassie dye with varying efficiency, this result is closer to IBD results.

The difference of iST was also highlighted in the pairwise comparisons of average LFQ intensities, where iST overall shows the lower coefficient of determination (*R* ^2^) and higher fold change variations (Figure S9). The lowest similarity observed in the pairwise comparisons (i.e., the minimum *R* ^2^ value) was found between the Strap and Pmax protocols. This result is expected, as these two protocols correspond to the samples with the highest and lowest total LFQ intensities, respectively (Figure 4).

To further investigate whether peptide composition influenced protein representation across the different protocols [32], we calculated the average isoelectric point (pI) and the grand average of hydropathicity (GRAVY) values for the identified corona proteins. The pI is the pH at which a protein carries no net electrical charge, and it influences protein solubility and interactions with surfaces. In turn, the GRAVY index reflects peptide hydrophobicity by averaging the hydropathy values of all amino acids in a sequence, with positive values indicating hydrophobic peptides and negative values indicating hydrophilic ones [35]. We computed the average GRAVY values and pI for each protocol, based on the 20 most abundant proteins. The results showed that all GRAVY values ranged from −0.14 to −0.17 and the pI values from 5.3 to 5.7 (Table S4). These values suggested that a great part of the corona is composed of positively charged amino acids, which are more attracted given the negatively charged NP surface. At the same time, the GRAVY values close to zero indicate a balanced mix of hydrophobic and hydrophilic amino acids, indicating a context‐dependent corona that is soluble in water but at the same time able to interact with hydrophobic surfaces to some degree, in line with previous literature [36]. Most importantly, no significant variation in either of the two parameters could be statistically found among the different protocols.

Shifting from quantitative comparisons to protein presence, UpSet plots were employed to assess both the similarities and unique detections across protocols (Figure 5C). Two different criteria were used to define protein presence. In the first approach, a protein was considered present if the average LFQ value across the four replicates was greater than zero. In the second, more stringent approach, a protein was considered present only if all four individual replicate LFQ values were non‐zero. The first method provides a broader overview of protein identification, while the second emphasizes reproducibility across replicates. Considering the first approach, 197 (44%) common proteins were identified (Figure 5C). The IBD protocol appears to be the one that differs most significantly from the others in terms of protein identification, as its exclusion resulted in the highest score of protein sharing (Figure 5C). Different protocols also feature unique proteins that are not shared with the others (Figure 5C). iST and Strap are showing the highest number of uniquely identified proteins. However, when the stringent protein identification approach was applied, the number of shared proteins dropped to 85 (27%) (Figure S8). Moreover, iST revealed the presence of 66 uniquely identified proteins.

## 4 Discussion

The physicochemical characterization of pristine and NP‐HCs confirmed the colloidal stability of both, an essential yet often overlooked prerequisite for obtaining reliable protein corona samples [29, 37]. The low PDI and consistent DCS and DLS profiles for both pristine and NP‐HCs further support their colloidal stability and monodispersity, key attributes for ensuring the accuracy of downstream proteomic analyses. The characterization of the obtained NP–HCs also confirmed the absence of protein aggregates that could contaminate the isolated protein corona, an often overlooked but important factor that should be considered [38]. The reproducibility of protein corona samples was confirmed by SDS‐PAGE, while protein quantification via microBCA was crucial for determining the appropriate trypsin amount and assessing compatibility with the working range of the selected protocols.

A comparative evaluation of digestion protocols highlighted substantial differences in performance, particularly in terms of processing time, handling complexity, peptide yield, digestion efficiency, and reproducibility. The IBD, while widely used and simple, exhibited the lowest proteomic performance, with a high proportion of missing LFQ values, the highest missed cleavage rates, likely due to suboptimal enzyme‐to‐protein ratios, although it registers the highest peptide‐to‐protein ratios, probably due to the low number of quantified proteins. The occurrence of intense late TIC peaks and corresponding high *m/z* signals without corresponding MS/MS identifications in the IBD replicates suggests incomplete digestion, a conclusion further supported by the low number of identified peptides and proteins.

The Pmax protocol, while time‐efficient, demonstrated the smallest reproducibility, as evidenced by high RSD values, low peptide and protein identification in replicates 2 and 4, and broad PCA clustering. This lower performance may be attributed to variable handling (e.g., vortexing steps) and low enzyme‐to‐protein ratio, reinforcing the need for standardized and robust workflows, especially when working with low‐abundance samples such as NP‐bound proteins. The other in‐gel digestion method including surfactant RapiG, showed better consistency.

The cartridge‐based protocols, Strap and iST, performed better in terms of identified proteins from in‐gel digestion protocols. Strap demonstrated a high peptide‐to‐protein ratio and a favorable LFQ‐based reproducibility profile, despite the challenge of meeting minimum protein input requirements, which may induce the high missed cleavage values. Notably, Strap was the only protocol to retain NP presence post‐processing, which may be responsible for the high LFQ values, having the particles potentially exposed to all the steps and therefore potentially more efficiently subjected to protein removal. Conversely, iST yielded the lowest missed cleavage values and a high number of uniquely identified proteins, likely due to its standardized system and inclusion of LysC alongside trypsin. iST's protein abundance profile deviated more from other protocols, with Apolipoprotein A‐I emerging as the most abundant protein instead of Histidine‐rich glycoprotein, a glycoprotein which was recognized to be the main component of HC‐SiNPs not only in the other tested protocols but also in previous studies [33, 39, 40].

It is well established that surfactants significantly affect the interaction of Apolipoprotein A‐I with nanoparticle (NP) surfaces, influencing both Apolipoprotein A‐I absorbance during corona formation [34] and its removal from previously formed corona [33]. In our in‐gel digestion protocols, all samples were first denatured using SDS‐DTT buffer and fully loaded into gel wells under identical electrophoretic conditions. Since even proteins tightly bound to NPs can migrate through the gel if NPs pellet is loaded in the well (as shown for graphene oxide [41]), the observed differences in Apolipoprotein AI abundance among IBD, RapiG, and Pmax protocols can be attributed to the presence or absence of surfactants during digestion, rather than a different removal from the NP surface. RapiG showed the lowest Apolipoprotein A‐I levels, consistent with previous findings [26], followed by IBD (no surfactant during digestion), and then Pmax, which gave the highest levels. This can be induced by surfactants’ varying abilities to denature proteins, which in turn alters the accessibility of proteolytic enzymes and ultimately the observed protein abundance [26]. Indeed, surfactants can both promote denaturation through electrostatic and hydrophobic interactions [34, 42] or, in some cases, stabilize protein structure and hinder digestion [43]. Therefore, it is possible that RapiG and Pmax induce opposite digestion effects, respectively reducing and increasing trypsin accessibility to Apolipoprotein AI compared to the value obtained in IBD, which is surfactant independent and indeed aligns more closely with the SDS‐PAGE result.

In cartridge‐assisted protocols, the higher Apolipoprotein A‐I content observed in both protocols likely reflects overall higher digestion efficiency, which results in a less competitive interaction with trypsin, or it can also be due to the more efficient removal of this protein from cartridges during the elution steps.

Multivariate analysis, including PCA and biplot interpretation, further reinforced protocol‐dependent clustering and variability, with iST and Strap yielding the most reproducible and distinct profiles. The influence of Apolipoprotein C‐IV on sample clustering emphasizes the importance of this class of proteins in driving the differences of these protocols. Protein overlaps and uniqueness analyses revealed further insights into protocol‐specific biases. When considering proteins present in all replicates (LFQ > 0), only 85 proteins (27%) were shared across protocols, underscoring the importance of stringent criteria for reliable protein identification. Notably, iST and Strap yielded the highest number of unique proteins under strict conditions, suggesting these methods enhance proteome coverage and may be preferable for comprehensive and reliable protein corona profiling. However, the effect of surfactant presence during digestion is a relevant parameter that has to be considered, particularly for the evaluation of protein relative abundance. This aspect was previously highlighted as relevant for bottom‐up proteomics [44, 45] but in the case of protein corona analysis is probably even more variable considering that it can also varying depending on NP characteristics [33].

In summary, while all tested protocols enabled identification of protein corona components, cartridge‐based protocols emerged as the most robust in terms of reproducibility, protein coverage, and digestion efficiency, with iST standing out for its speed and minimal hands‐on time. After all the protocols, we performed an additional C18 purification step, which may not be strictly necessary. The reason for introducing this step was to avoid the risk of NP presence in the final samples; however, skipping it could further streamline the process and increase protein recovery. Nowadays, the drawback of cartridge‐based protocols is the significantly higher cost. For more cost‐effective workflows, the in‐gel digestion protocol remains a viable option, especially when optimized with surfactants and an appropriate trypsin‐to‐protein ratio. Regarding the Pmax protocol, better sample handling, such as using a shaker instead of vortexing, as recommended for processing larger sample batches, could improve reproducibility and overall performance.

This study presents a comprehensive evaluation of five different proteomic sample preparation protocols for characterizing the HC of NPs. The results reported in this article clearly indicate that, while no single method can be ranked as the best across all metrics, each protocol introduces distinct advantages and limitations that influence the outcome of NP‐protein corona characterization. Therefore, the selection of the most appropriate protocol should be tailored to the specific analytical objective, such as maximizing proteome coverage, reproducibility, throughput, or cost‐efficiency and nanoparticles characteristics. Indeed, nanoparticle physicochemical properties can deeply affect the outcome of proteomics protocols. Therefore, the extrapolation of the current results to particles different from the one used in this study should be made with caution. Nevertheless, the central finding that sample preparation workflows strongly influence protein corona proteomics outcomes is expected to be broadly applicable across nanoparticle types. This has broad implications, as the identification of specific proteins within the NP corona, for example, in the context of biomarker discovery, can be strongly influenced by the chosen sample preparation protocol and, consequently, draw conclusions which may not be directly comparable with the real scenario. The divergence in protein profiles highlights a critical issue in the field: the lack of standardized proteomic workflows significantly contributes to the discrepancies observed in inter‐laboratory studies, even when analyzing the same sample type. Such inconsistencies can be further incremented by variations in computational approaches used for data processing and interpretation [9, 46]. These findings emphasize the urgent need for standardized protocols in NP‐protein corona research, particularly within the proteomics area [7]. Standardization is essential not only for ensuring reproducibility but also for advancing our collective understanding of nano‐bio interactions in accordance with the FAIR (findable, accessible, interoperable, and reusable) data principles. This includes clear documentation of sample preparation steps, transparent reporting of analytical conditions, and adoption of consensus criteria for protein identification and quantification. We believe that establishing community‐driven best practices for sample preparation and NP corona proteomics data analysis will be a crucial step toward enabling reproducible, high‐quality, and comparable data. This initiative would not only improve the field but also facilitate the integration of datasets across laboratories and enhance the reliability of predictive models, such as those employed in QSAR studies and biomarker discovery initiatives.

## Author Contributions

**Asia Saorin**: conceptualization, validation, investigation, visualization, formal analysis, methodology, writing – original draft, review & editing. **Alberto Martinez‐Serra**: conceptualization, validation, investigation, visualization, formal analysis, methodology, writing – original draft, review & editing. **Michael Henry**: investigation, methodology, writing – review & editing. **Paula Meleady**: methodology, writing – review & editing. **Marco P. Monopoli**: conceptualization, supervision, funding acquisition, project administration, methodology, writing – review & editing.

## Funding

This project was supported by the European Union's Research and Innovation Programmes under grant agreement no. 101092901 (POTENTIAL). The Orbitrap Fusion Tribrid mass spectrometer was funded by a Science Foundation Ireland Infrastructure Award to Dublin City University (SFI 16/RI/3701).

## Conflicts of Interest

The authors declare no conflicts of interest.

## Supporting information



The Supporting Information contains a Table with the details of the applied protocols and the supplementary materials.
**Supporting File 1**: pmic70118‐sup‐0001‐SuppMat.docx.

**Supporting File 2**: pmic70118‐sup‐0002‐ProtocolTable.docx.

## Data Availability

The data that support the findings of this study are openly available in Zenodo at https://zenodo.org/uploads/16813857, reference number 10.5281/zenodo.16813857. The mass spectrometry proteomics raw data and Max Quant results (txt files) have been deposited to the Zenodo repository at the link 10.5281/zenodo.16813856.
