# Supplementary material for: Mass Spectrometry Proteomics of the Nanoparticle Corona Is Highly Dependent on Sample Preparation Protocol
Source: Proteomics. 2026 Mar 27;26(5):98–111. doi: 10.1002/pmic.70118 (PMC13106918; doi:10.1002/pmic.70118)
Supplement: Supplementary file 1 — The Supporting Information contains a Table with the details of the applied protocols and the supplementary materials. Supporting File 1: pmic70118‐sup‐0001‐SuppMat.docx. [file PMIC-26--s001.docx]

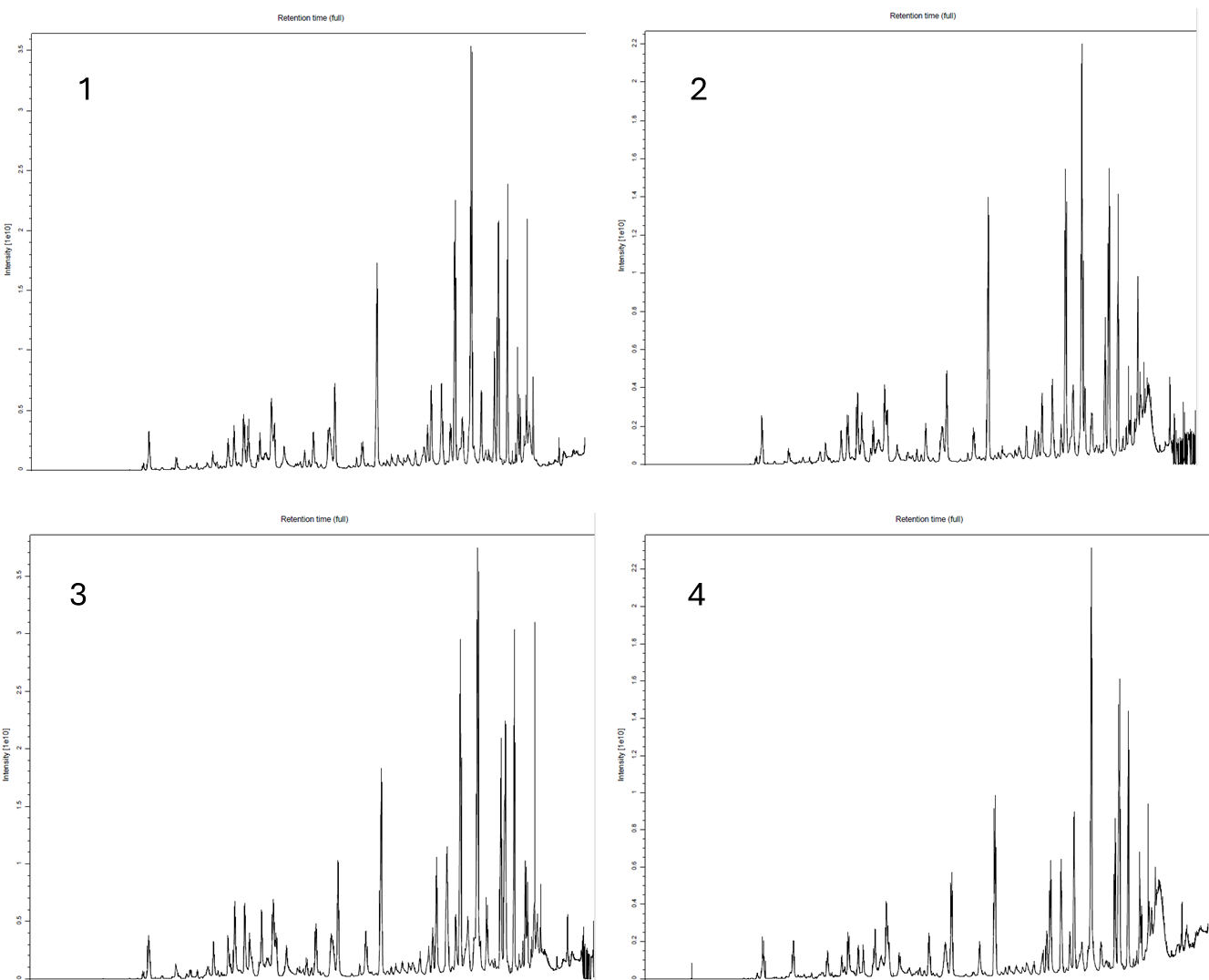


Supplementary Figure 1 Total Ion Chromatogram (TIC) of the four replicates of iST


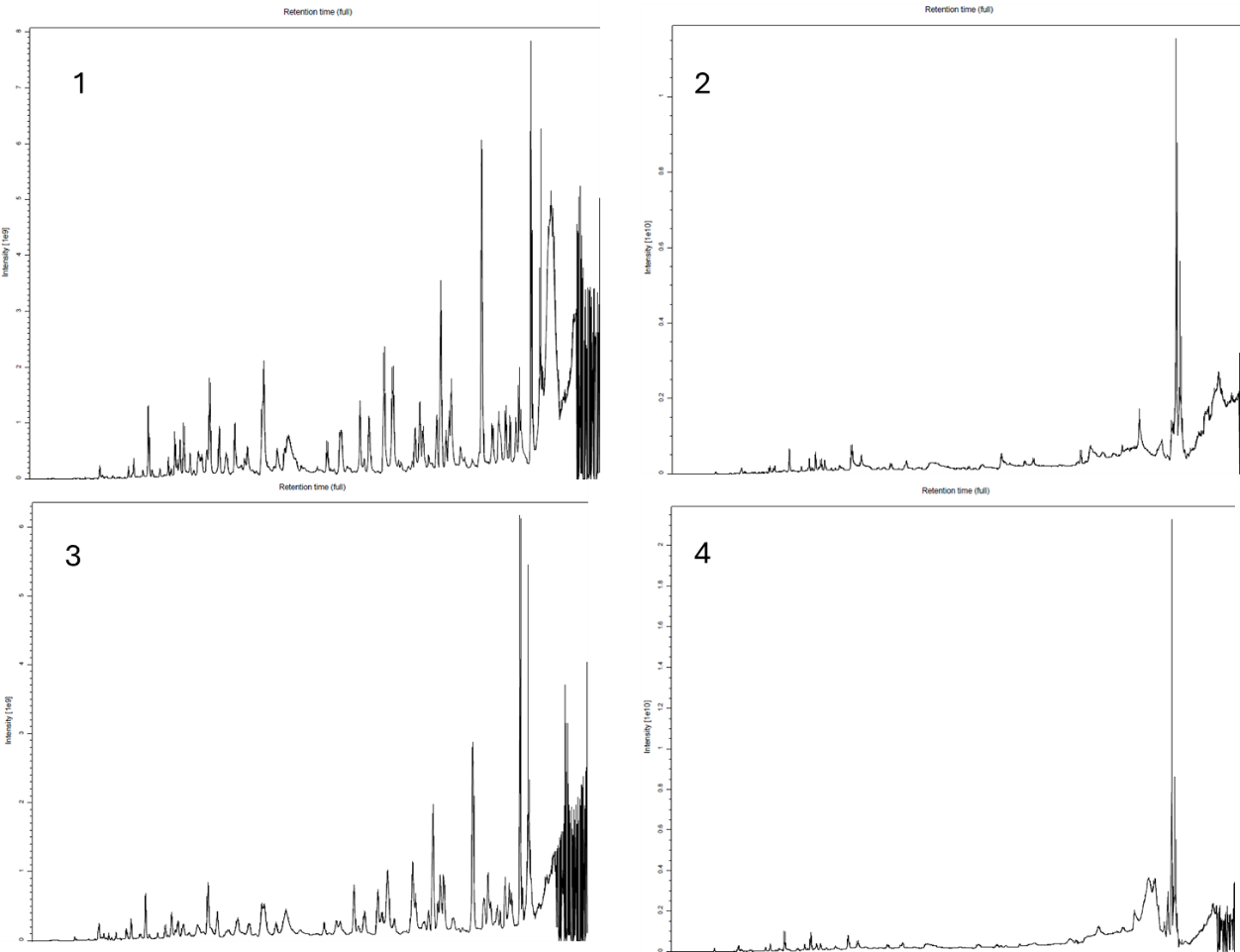


Supplementary Figure 2 Total Ion Chromatogram (TIC) of the four replicates of ProteasMAX


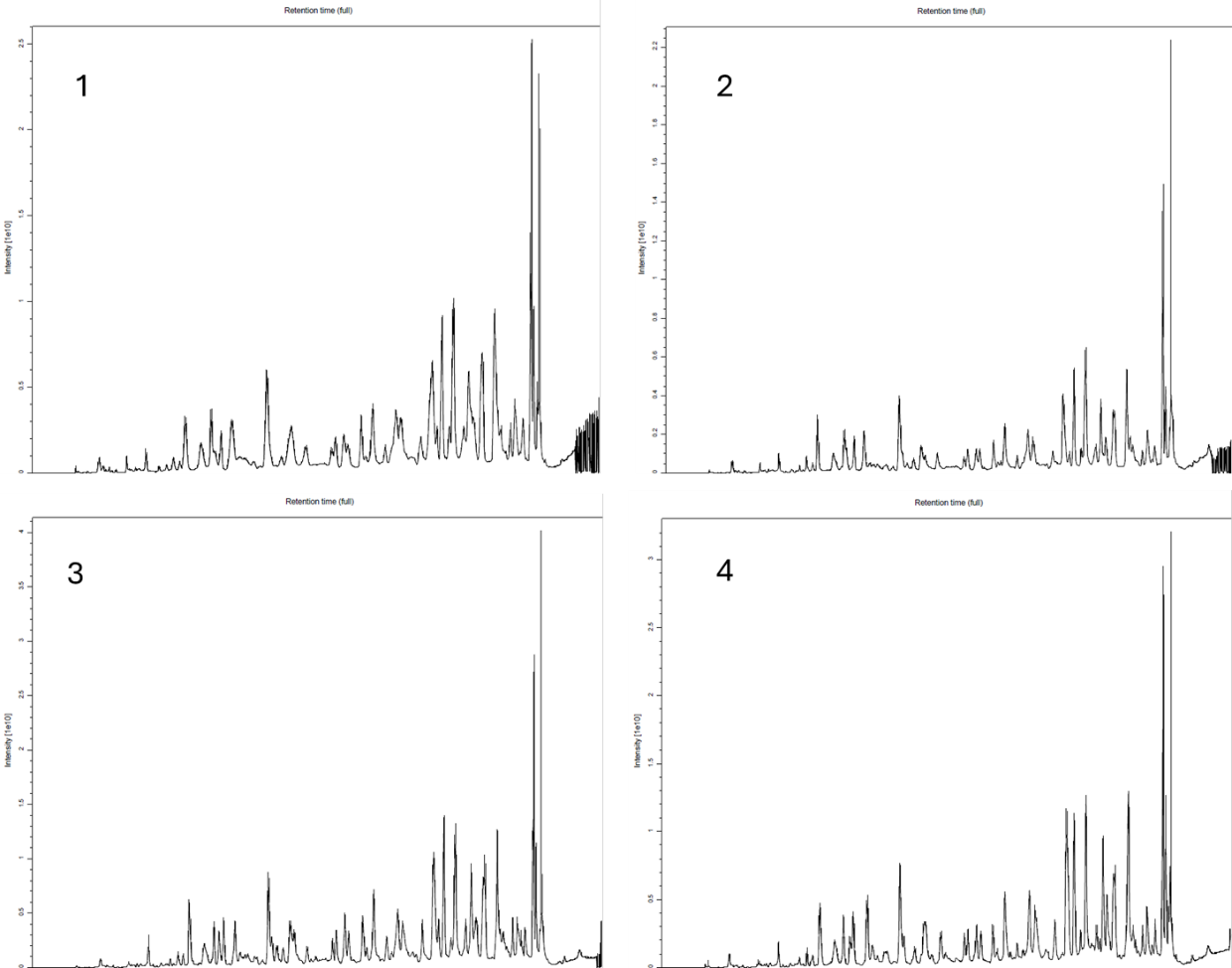


Supplementary Figure 3 Total Ion Chromatogram (TIC) of the four replicates of RapidGest


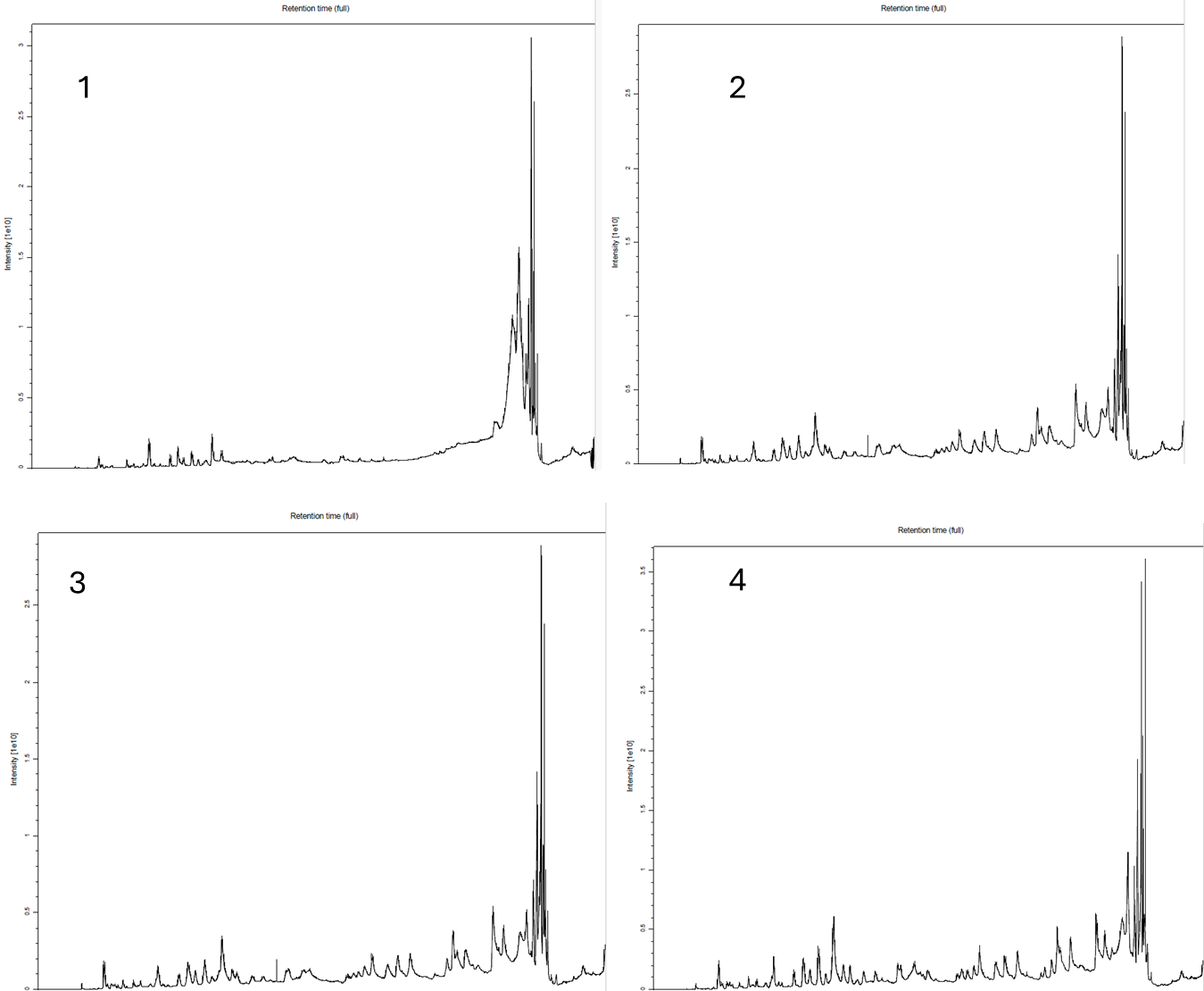


Supplementary Figure 4 Total Ion Chromatogram (TIC) of the four replicates of IBD


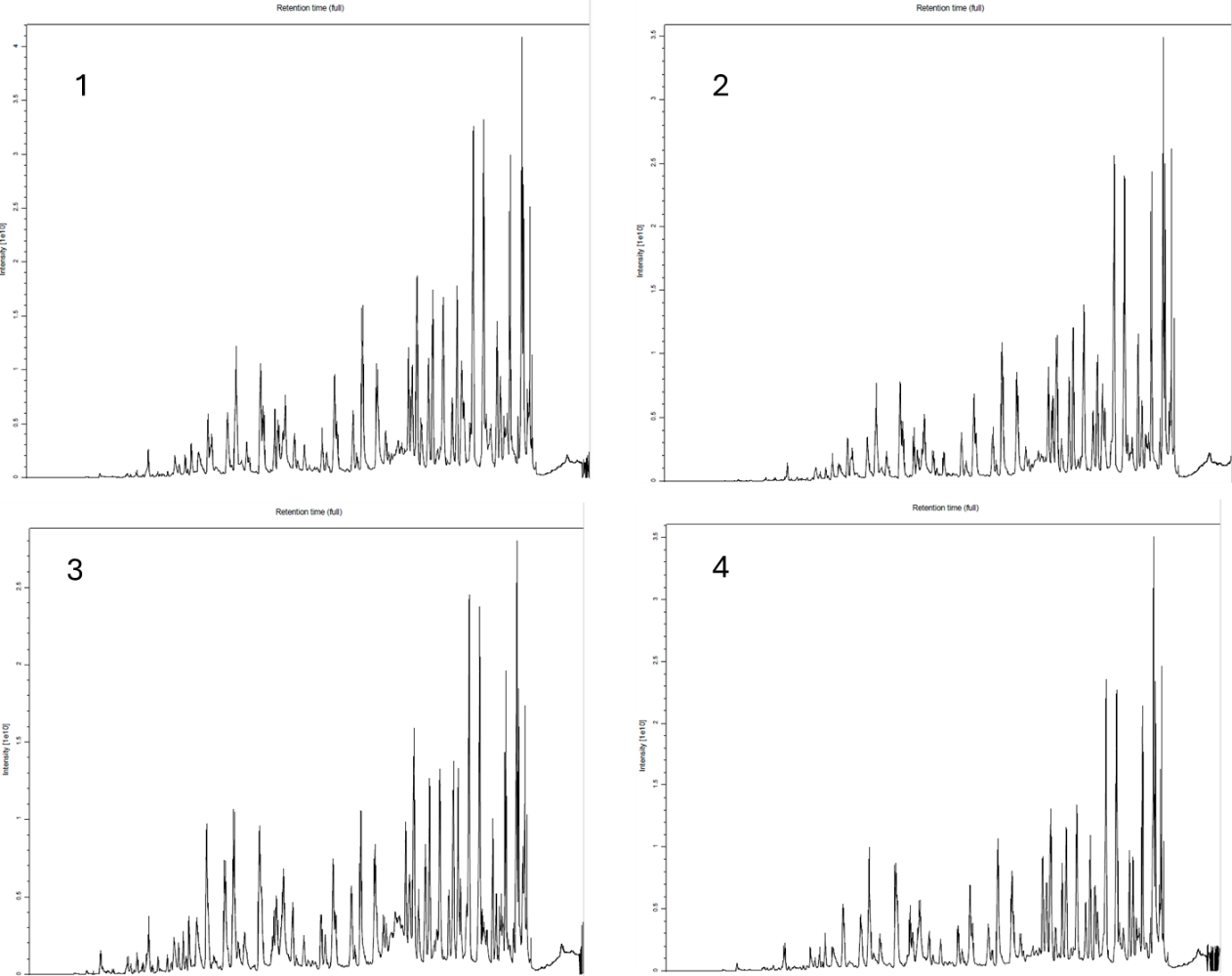


Supplementary Figure 5 Total Ion Chromatogram (TIC) of the four replicates of S-Trap

Supplementary Figure 6 Fractions of high-mass precursors (m/z ≥ 800) in the 50–60 min retention time window for different proteomic sample preparation methods. Boxplots represent the distribution of intensity fractions across replicates. Statistical differences between methods are indicated by letters above the boxes (ANOVA with post-hoc test, p < 0.05). Higher fractions indicate a larger proportion of undigested or long peptides.

Supplementary Table 1Proteins and peptides counts for the replicates of each protocol

|  | **Proteins (LFQ>0)** | | | | **Peptides (MS/MS>0)** | | | |
| --- | --- | --- | --- | --- | --- | --- | --- | --- |
| **Protocol** | **R1** | **R2** | **R3** | **R4** | **R1** | **R2** | **R3** | **R4** |
| **iST** | 322 | 342 | 307 | 342 | 2751 | 2604 | 2588 | 2610 |
| **Pmax** | 367 | 186 | 336 | 126 | 3122 | 1735 | 2426 | 1646 |
| **RapiG** | 254 | 344 | 293 | 288 | 2734 | 3128 | 3150 | 3064 |
| **IBD** | 115 | 139 | 221 | 148 | 2113 | 2307 | 2647 | 2745 |
| **Strap** | 258 | 293 | 263 | 283 | 3318 | 3175 | 3413 | 3283 |

Supplementary Table 2 Average proteins and peptides counts and their ratio in each protocol (error refers to SD)

| **Protocol** | **Proteins (LFQ>0)** | **Peptides (MS/MS>0)** | **peptides/proteins** |
| --- | --- | --- | --- |
| **iST** | 328±14 | 2638.2±75.7 | 8±0.4 |
| **Pmax** | 253±100 | 2232.2±688 | 9±4 |
| **RapiG** | 294±32 | 3019±193.5 | 10±1 |
| **IBD** | 155±39 | 2453±294.3 | 16±4 |
| **Strap** | 274±14 | 3297.2±98.3 | 12±0.7 |


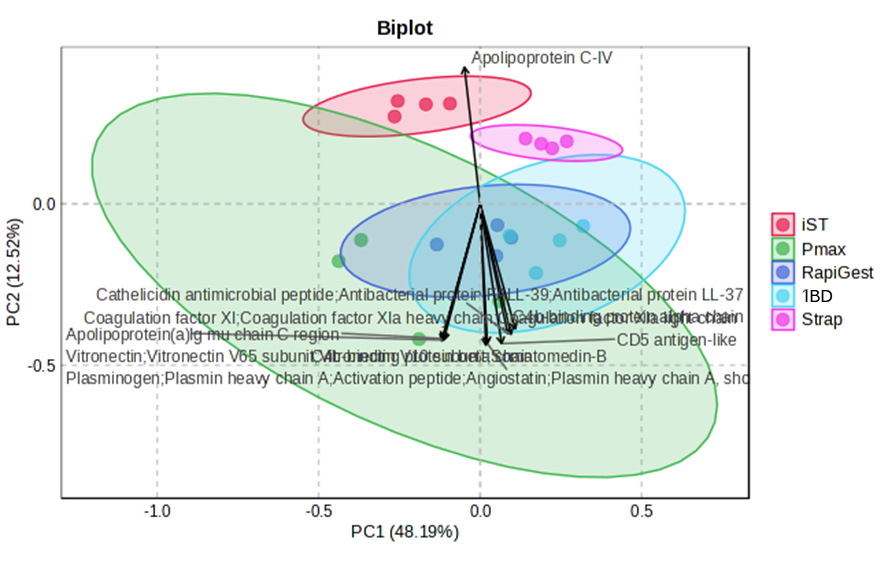


Supplementary Figure 7 PCA biplot between the selected PCs with highlighted proteins driving the separation.

Supplementary Table 3. Top 20 proteins in each protocol. Reported values refer to protein percentage on total LFQ value.

| **Protein names** | **iST** | **Pmax** | **RapiG** | **IBD** | **Strap** |
| --- | --- | --- | --- | --- | --- |
| Actin, cytoplasmic 1;Actin, cytoplasmic 1, N-terminally processed;Actin, cytoplasmic 2;Actin, cytoplasmic 2, N-terminally processed | x | 0.44 | x | x | x |
| Alpha-1-antitrypsin;Short peptide from AAT | 0.45 | x | 0.28 | 0.26 | 0.21 |
| Apolipoprotein A-I;Proapolipoprotein A-I;Truncated apolipoprotein A-I | 52.4 | 24.96 | 10.12 | 18.4 | 29.53 |
| Apolipoprotein A-II;Proapolipoprotein A-II;Truncated apolipoprotein A-II | 5.31 | 2.98 | 0.37 | 3.12 | 1.46 |
| Apolipoprotein B-100;Apolipoprotein B-48 | 0.89 | 1.07 | 0.77 | 0.77 | 0.5 |
| Apolipoprotein C-I;Truncated apolipoprotein C-I | 0.3 | x | x | x | 1.02 |
| Apolipoprotein C-II;Proapolipoprotein C-II | 0.6 | x | x | x | x |
| Apolipoprotein C-III | 1.39 | x | x | x | 0.18 |
| Apolipoprotein E | 0.97 | 1.02 | 0.37 | 0.52 | 0.49 |
| Coagulation factor XI;Coagulation factor XIa heavy chain;Coagulation factor XIa light chain | 0.41 | 1.38 | 0.9 | 1.38 | 0.62 |
| Fibrinogen beta chain;Fibrinopeptide B;Fibrinogen beta chain | 0.44 | x | 0.52 | 0.21 | 0.36 |
| Fibrinogen gamma chain | 0.42 | 0.53 | 0.62 | 0.39 | 0.36 |
| Heat shock cognate 71 kDa protein | 0.3 | 0.54 | x | x | x |
| Histidine-rich glycoprotein | 24.72 | 32.29 | 61.17 | 52.17 | 45.15 |
| Histone H2A type 1-J;Histone H2A type 1-H;Histone H2A.J;Histone H2A type 1-C;Histone H2A type 3;Histone H2A type 1-D;Histone H2A type 1;Histone H2A type 1-B/E;Histone H2AX;Histone H2A type 1-A | x | 0.8 | x | x | x |
| Ig alpha-1 chain C region | x | 0.43 | 0.25 | 0.3 | 0.15 |
| Ig gamma-1 chain C region | x | x | 0.2 | x | x |
| Ig kappa chain C region | 0.32 | 0.82 | 0.34 | 0.37 | 0.26 |
| Ig lambda-6 chain C region | 0.38 | 0.43 | x | 0.25 | x |
| Ig mu chain C region | x | x | 0.21 | 0.24 | x |
| Inosine-5-monophosphate dehydrogenase 2 | x | 0.59 | x | x | x |
| Keratin, type II cytoskeletal 1 | x | 0.56 | x | 0.22 | 0.17 |
| Kininogen-1;Kininogen-1 heavy chain;T-kinin;Bradykinin;Lysyl-bradykinin;Kininogen-1 light chain;Low molecular weight growth-promoting factor | 2.29 | 17.2 | 15.96 | 14.11 | 14.23 |
| Plasma kallikrein;Plasma kallikrein heavy chain;Plasma kallikrein light chain | 1.02 | 1.3 | 0.62 | 1.08 | 0.52 |
| Plasma protease C1 inhibitor | 0.32 | x | 0.39 | 0.47 | 0.37 |
| Plasminogen;Plasmin heavy chain A;Activation peptide;Angiostatin;Plasmin heavy chain A, short form;Plasmin light chain B | 0.32 | 0.99 | 0.84 | 0.77 | 0.5 |
| Prothrombin;Activation peptide fragment 1;Activation peptide fragment 2;Thrombin light chain;Thrombin heavy chain | x | x | 0.19 | x | x |
| Serum albumin | 0.43 | 0.69 | 0.49 | 0.49 | 0.31 |
| Vitronectin;Vitronectin V65 subunit;Vitronectin V10 subunit;Somatomedin-B | x | 0.51 | 0.35 | 0.35 | 0.23 |
| **Total % of top 20 proteins** | 91.09 | 87.16 | 94.57 | 95.87 | 95.42 |


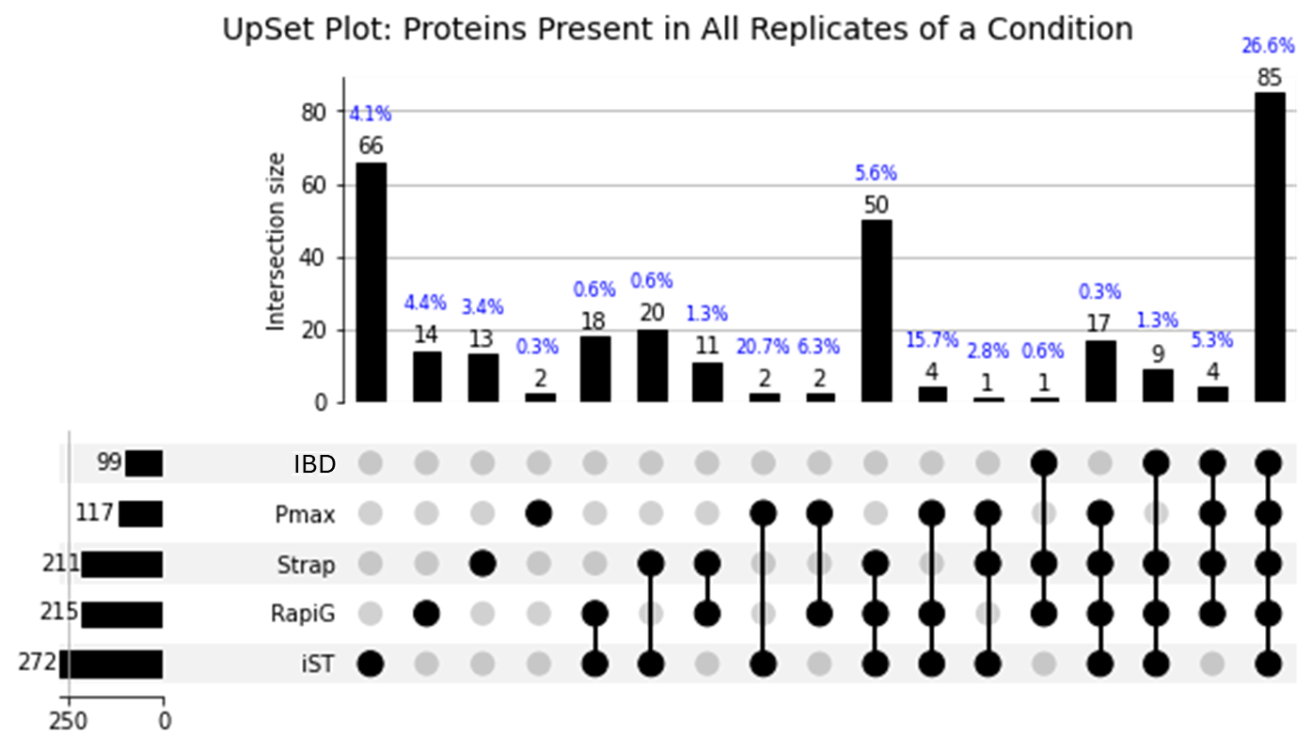


Supplementary Figure 8 UpSet plot analysis of protein presence filtered for presence in all four replicates (LFQ>0 for all).


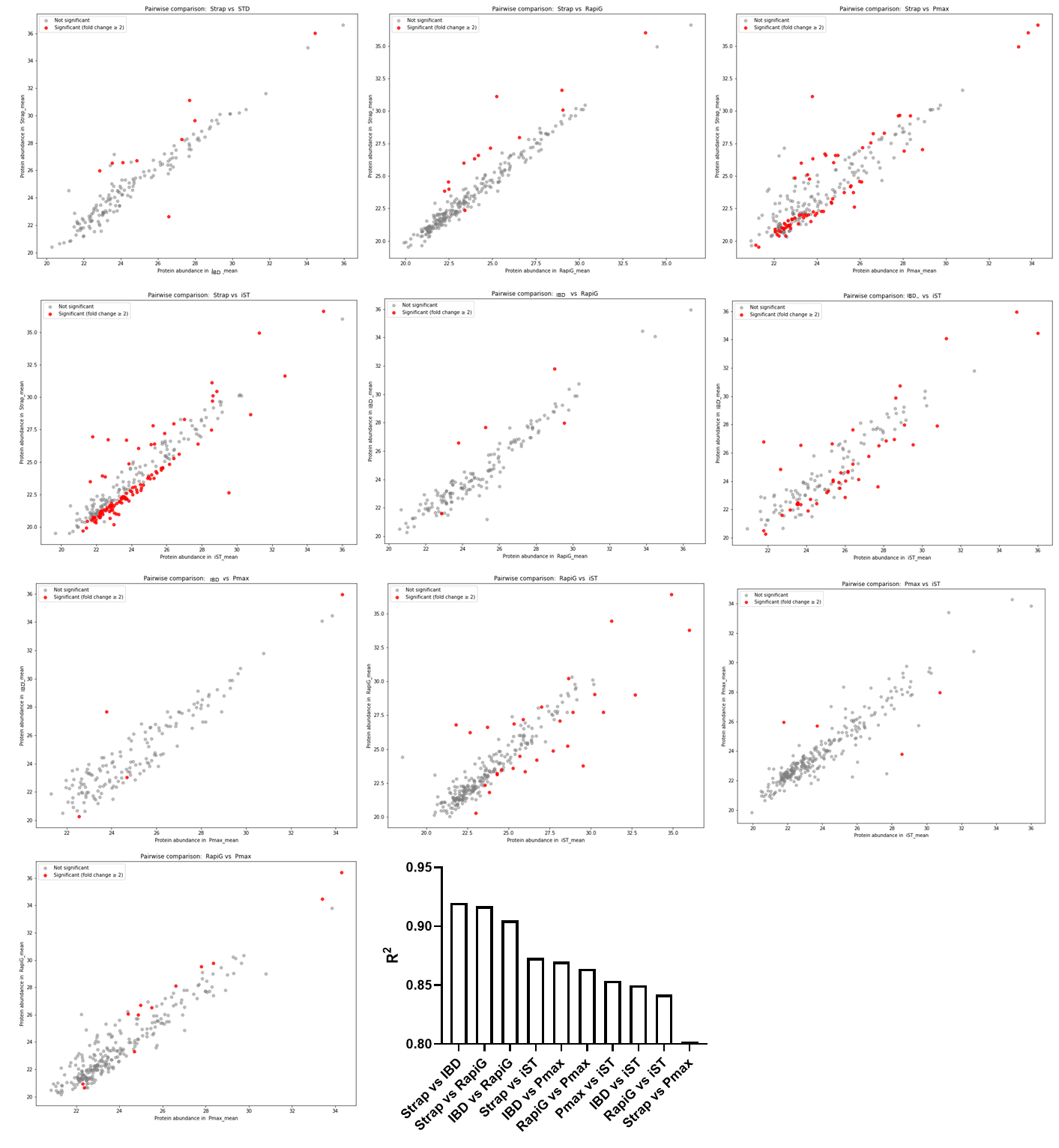


Supplementary Figure 9 Pairwise comparison of LFQ values between protocols. Proteins showing at least a twofold change in abundance are highlighted. Bar plots reports the R^2^ values of comparions
