# Supplementary material for: Mass Spectrometry Proteomics of the Nanoparticle Corona Is Highly Dependent on Sample Preparation Protocol
Source: Proteomics. 2026 Mar 27;26(5):98–111. doi: 10.1002/pmic.70118 (PMC13106918; doi:10.1002/pmic.70118)
Supplement: Supplementary file 2 — Supporting File 2: pmic70118‐sup‐0002‐ProtocolTable.docx. [file PMIC-26--s002.docx]

Table: details of applied protocols

| **Step** | **STD Protocol** | **Rapigest** | **ProteaseMAX** | **S-Trap** | **iST Kit** |
| --- | --- | --- | --- | --- | --- |
| **protein required (ug)** | None | None | Minimum 50 ng | 100-300* | 1-100 |
| **Sample preparation** | Loading buffer (SDS, DTT)-95°C, 5 min, gel run | | | NA | NA |
|  | Gel area cut in 4 | Gel area cut smaller than 1 mm³ | |  |  |
| **Washing** | Multi-step with ACN/NH₄HCO₃; ~40 min | Multi-step with water, 50% ACN, 100% ACN; ~60 min | Multi-step with water, MeOH/NH₄HCO₃, ACN; ~ 20 min | Lysis buffer-5 min | NA |
| **Reduction** | 10 mM DTT, 100 µL, 56°C, 60 min | 10 mM DTT, 50 µL, 56°C, 45 min | 25 mM DTT, 100 µL, 56°C, 20 min | 2 µL of 120 mM DTT, 55°C, 15 min | LYSE buffer-95°C, 10 min |
| **Alkylation** | 50 mM IAA, 100 µL, RT, dark, 30 min | 55 mM IAA, 60 µL, RT, dark, 30 min | 55 mM IAA, 100 µL, RT, dark, 20 min | 2 µL of 500 mM IAA, RT, dark, 10 min (+acidification) |  |
| **Digestion buffer** | **Trypsin gold** in 50 mM NH₄HCO₃ | **Trypsin gold** in NH₄HCO₃ + RapiGest | **Trypsin gold** + 0.01% ProteaseMAX-50mM NH₄HCO₃ | ****Trypsin gold** in 50mM NH₄HCO₃ | DIGEST buffer (Trypsin/**LysC**) |
| **Trypsin V, [C]** | 100 µL, 1:50 try/prot | 30 µL ~ 1:10 try/prot | 20µL trypsin (1:20 try/prot)+80 µL( buffer) | 125 µL-1:10 try/prot | 50 µL (unknown concentration) |
| **Digestion** | Overnight, 37°C (~16 h) | Overnight, 37°C (~16 h) | 1 h at 50°C* | Overnight, 37°C ~16 h (alternatively 47°C 1-2h)^‡^ | 1 h at 37°C |
| **Post-digestion cleanup** | ACN/TFA Multi-step ~20min  dry | TFA 0.5%, centrifuge*  dry | TFA 0.5%, ice-centrifuge**  dry | Tris+ formic acid/ACN, water Multi-step ~5min  dry*** | WASH 1+2, ELUTE- ~10min  *dry |
| **Total Time Estimate** | ~18–20 h | ~18–20 h | ~2-3 h | ~18–20 h (could be reduced)^‡^ | ~3 h |
| **note** |  | * no pellet was observed | *Samples present different volume at the end of the incubation. In particular n°4 was dry  **this step was added to remove the ProteasMAX but no pellet was observed | *out of range  **Not respected the minimum of 10 ug of trypsin  ***NPs detected | *LC-LOAD buffer was not used for final reconstitution. Samples followed common C18 tips protocols |
